# The Transcription Factor FgSge1 Harnesses the SAGA Complex to Activate Mycotoxin Biosynthesis and Fungal Virulence

**DOI:** 10.1002/advs.202518558

**Published:** 2026-03-12

**Authors:** Yueqi Zhang, Jingrui Wang, Qiaowan Chen, Chen Jiao, Yun Chen, Zhongshou Wu, Zhonghua Ma

**Affiliations:** ^1^ National Key Laboratory of Rice Biological Breeding Zhejiang University Hangzhou China; ^2^ Institute of Biotechnology Key Laboratory of Molecular Biology of Crop Pathogens and Insects Zhejiang University Hangzhou China; ^3^ Yazhouwan National Laboratory Sanya China

**Keywords:** chromatin conformation, *Fusarium graminearum*, histone acetylation, mycotoxin, transcription factor

## Abstract

Understanding how fungi regulate mycotoxin production is critical for managing crop diseases and reducing contamination in food systems. Here, we elucidate the mechanism of the transcription factor FgSge1 in *Fusarium graminearum*, a significant fungal pathogen responsible for Fusarium head blight in cereal crops, in regulating mycotoxin biosynthesis and pathogenicity. FgSge1 specifically binds to the 8‐bp cis‐element TAARGTTT. Under mycotoxin‐induced conditions, FgSge1 binds to this cis‐element within its own promoter, activating its own transcription. Additionally, FgSge1 connects with this cis‐element within the promoters of mycotoxin biosynthesis genes and interacts directly with the scaffold protein FgAda2 of the Spt‐Ada‐Gcn5‐Acetyltransferase (SAGA) complex. This interaction recruits the histone acetyltransferase FgGcn5 to the promoters of DON biosynthetic genes, promoting histone acetylation and facilitating jet‐like chromatin architecture, thereby activating transcription. In contrast, the FgSge1 mutant fails to recruit the SAGA complex, leading to reduced histone acetylation, disrupted chromatin structure, and impaired DON biosynthesis. Together, these findings establish FgSge1 as a critical self‐activating regulatory factor that links histone acetylation to higher‐order chromatin remodeling, orchestrating mycotoxin gene activation, and providing a framework for understanding epigenetic control of mycotoxin biosynthesis and virulence in fungi.

## Graphical abstract

This study reveals the molecular mechanism by which the transcription factor FgSge1 regulates mycotoxin biosynthesis and virulence in *Fusarium graminearum*. FgSge1 binds to the TAARGTTT cis‐element, enabling self‐activation. It recruits the SAGA complex, promotes histone acetylation, and facilitates jet‐like chromatin remodeling, thereby activating target gene expression. This work demonstrates how FgSge1 links epigenetic modification to chromatin reorganization in mycotoxin regulation.

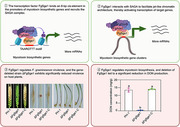

## 1 Introduction

*Fusarium graminearum* is a major pathogenic fungus responsible for Fusarium head blight (FHB), a devastating disease affecting cereal crops worldwide. FHB not only causes significant yield losses but also contaminates grains with harmful mycotoxins, particularly deoxynivalenol (DON), which pose serious health risks to humans and animals [1]. DON biosynthesis is governed by a cluster of at least 15 *TRI* genes dispersed across the genome, whose expression is tightly regulated by environmental cues and multiple transcription factors [1]. Key transcription factors such as Tri6, Tri10 [2], FgAreA [3], FgPacC [4], FgStuA [5], and FgSge1 [6] coordinate the expression of these *TRI* genes, with many knockout mutants affecting both mycotoxin production and fungal development, highlighting the intricate regulatory complexity [1, 6].

Among these transcription factors, FgSge1, homologous to Wor1 in *Candida albicans*, has been identified as a central factor controlling both virulence and DON biosynthesis in *F. graminearum* [6, 7, 8]. Sge1 has also been characterized in other fungal pathogens, where it influences effector secretion and virulence [9, 10]. Notably, studies by Jonkers et al. demonstrated that deletion of FgSge1 does not significantly affect mycelial growth but severely attenuates the virulence of the strain on wheat heads and completely inhibits DON biosynthesis [6], which is partially attributed to its phosphorylation and regulatory role on the expression of the *TRI* genes [6, 8]. Despite its recognized importance, the molecular mechanisms by which Sge1 regulates secondary metabolism and virulence remain incompletely understood.

In eukaryotes, the SAGA complex plays a pivotal role in transcription regulation through its histone acetyltransferase (HAT) activity, primarily mediated by the histone acetyltransferase Gcn5 [11]. Dysfunction of Gcn5 has been linked to various human diseases, including neurological disorders, cancers, and autoimmune disorders. In plants, *Arabidopsis* Gcn5 has been implicated in embryogenesis, root growth, leaf and flower development [12, 13]. Mechanically, the multi‐protein complex interacts with transcription factors, recruiting Gcn5 via cofactors such as Ada2, to modify histones and alter chromatin accessibility [13, 14, 15]. For instance, *Arabidopsis* transcription factor bZIP11 recruits Gcn5 to the auxin‐responsive gene through coactivator Ada2b, thereby activating the transcription of corresponding genes [15]. In fungi, the SAGA complex influences numerous biological processes, including stress responses, development, and secondary metabolism [16, 17]. Deletion of Gcn5 in *F. graminearum* significantly reduces DON production and virulence, correlating with impaired histone acetylation and the disruption of jet‐like chromatin architecture associated with active gene transcription [18]. These observations underscore the importance of SAGA‐mediated chromatin remodeling in fungal virulence and mycotoxin biosynthesis, but the mechanisms linking SAGA to specific gene activation remain unclear.

In this study, we demonstrate that FgSge1 binds to cis‐elements within its own promoter, activating its transcription in response to toxin‐induced conditions. Notably, FgSge1 binds to the cis‐regulatory elements in the promoters of secondary metabolic biosynthesis gene clusters (SMBC), activating their transcription. FgSge1 interacts directly with the transcriptional coactivator FgAda2, facilitating the recruitment of the histone acetyltransferase FgGcn5 to target gene promoters. This recruitment promotes histone acetylation, which is essential for establishing jet‐like chromatin structures characteristic of active SMBC loci. Loss of FgSge1 reduces histone acetylation and diminishes these chromatin structures, leading to decreased secondary metabolite gene expression and mycotoxin production. Our findings reveal a novel mechanism whereby a fungal transcription factor integrates histone acetylation and chromatin architecture to regulate secondary metabolism and virulence.

## 2 Results

### 2.1 FgSge1 Specifically Regulates DON Production and Virulence in *F. graminearum*

A previous study indicates that FgSge1, also referred to as FgFgp1, is essential for *F. graminearum* DON biosynthesis and virulence [6]. Notably, the Gti1/Pac2 transcription factor FgSge1 is fungi‐specific [16], it is broadly conserved across fungal lineages, with only a few rare exceptions where homologs are not detected (Figure S1). Consistent with the findings of Jonkers et al., our results demonstrated that deletion of the *FgSGE1* gene does not significantly affect mycelial growth (Figure S2A), but it results in a marked reduction in DON production (Figure S2B). Furthermore, the mutant lost the virulence on wheat spikes, wheat coleoptiles, and maize silks (Figure S2C–E). To assess potential cell wall stress in the FgSge1 mutants, we evaluated their sensitivity and observed no significant sensitivity differences compared to the wild‐type PH‐1 strain (Figure S2F). Given that mycelial penetration is a critical virulence step for virulence, we further examined the penetration ability of the ΔFgSge1 mutant using the cellophane method. The penetration ability of the mutants showed no significant change compared to the wild‐type PH‐1 (Figure S2G). Additionally, we analyzed the expression levels of penetration‐related genes, including those responsible for galactosidase and glucosidase synthesis, glycoside hydrolase, lipase releasing free fatty acid, xyloside xylohydrolase, and glucosidase. Our findings indicated no significant transcriptional changes in these genes when compared to PH‐1 (Figure S2H). Taken together, these results suggest that FgSge1 specifically regulates DON production and virulence in *F. graminearum*, without directly participating in the regulation of other fundamental processes such as mycelial growth or penetration.

### 2.2 Identification of DNA Binding Sites and cis‐elements for FgSge1

To further investigate the function of FgSge1, we examined its transcriptional pattern and subcellular localization. The strain ΔFgSge1::Sge1‐GFP, expressing a GFP‐tagged FgSge1 in ΔFgSge1 background, was cultured in MM and TBI medium. In MM, the FgSge1‐GFP predominantly localized in the nucleus with weak fluorescence; while in TBI medium, its fluorescence intensity was significantly higher (Figure S3A). Additionally, the transcriptional levels of *FgSGE1* in the wild‐type strain PH‐1 were approximately 7‐fold higher in TBI compared to that in MM (Figure S3B). These findings indicate that FgSge1 primarily resides in the nucleus and is strongly induced in TBI medium, correlating with increased production of metabolic compounds.

**FIGURE 1:**
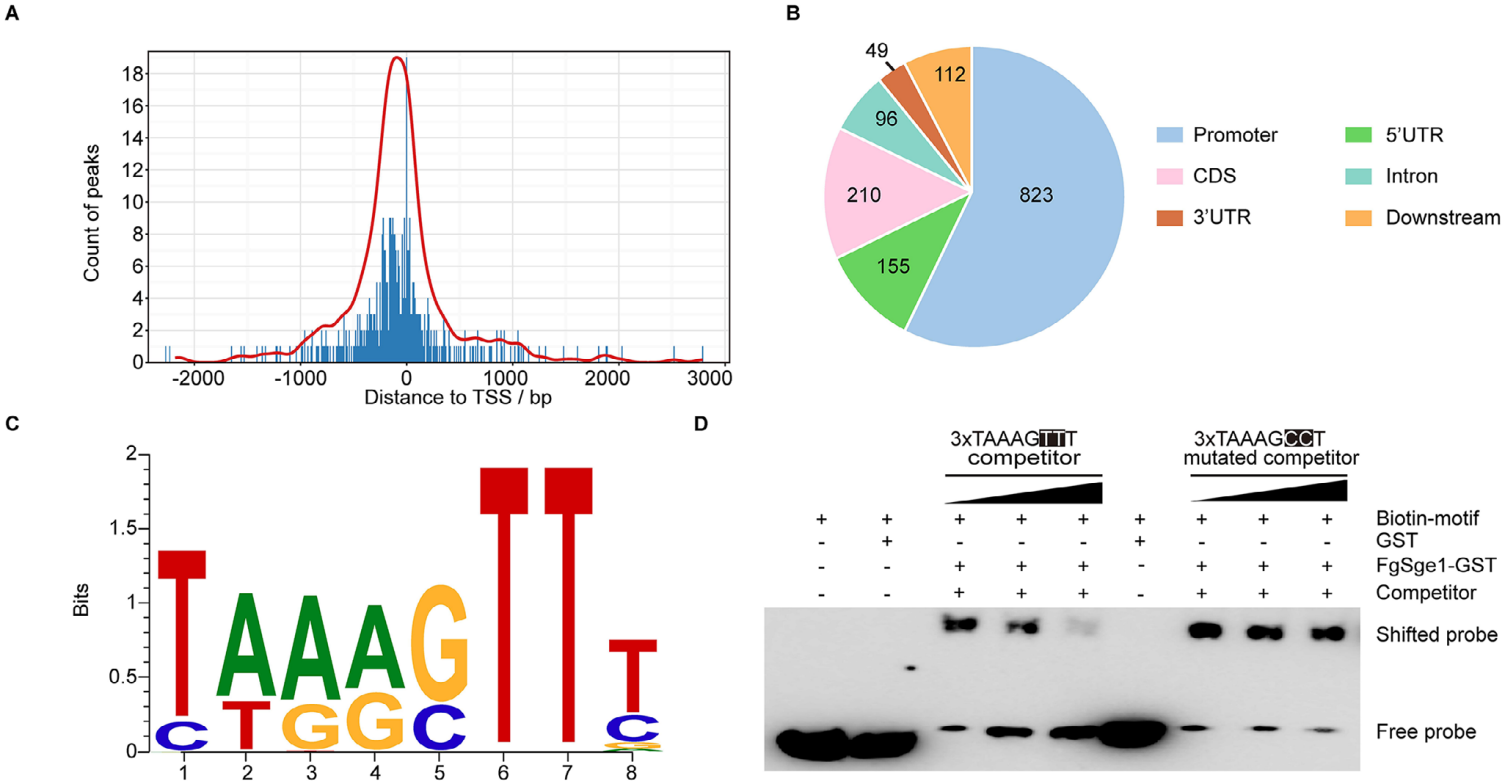
Analysis of FgSge1 binding sites and specific motif. (A) The FgSge1‐binding peaks are highly enriched within 1‐kb upstream regions of transcription start site (TSS). (B) Distributions of a total of 1445 FgSge1‐binding sites in promoter, coding sequence (CDS), intron, downstream, 5’ untranslated region (UTR), and 3’UTR. Data analysis combined with two biological replicates. (C) The putative cis‐element obtained by analyzing sequences of FgSge1‐binding peaks with the Sensitive, Thorough, Rapid, Enriched Motif Elicitation (STREME) program. (D) Verification of the binding of FgSge1 with the cis‐element by electrophoretic mobility shift assay (EMSA). The wild type (WT) DNA fragment consisting of three repeats of the cis‐element was labeled with biotin. The unlabeled WT or mutated DNA fragments with a concentration of 50, 100, or 150‐folds of unlabeled DNA fragment were used as competitors. The mutated nucleotides are shaded in black.

Given its role as a transcription factor, we performed ChIP‐seq experiments under TBI conditions to identify FgSge1 target genes. The results revealed that FgSge1 preferentially binds to promoter regions, particularly within 1000 bp upstream of the transcription start site (TSS) (Figure 1A,B). Further motif analysis with STREME identified an 8‐bp cis‐element (TAARGTTT) as the most probable binding site (Figure 1C). To validate this, electrophoretic mobility shift assays (EMSA) and probe competition binding assays were conducted. EMSA assay confirmed that FgSge1 directly binds a 24‐bp DNA fragment containing three repeats of this cis‐element (Figures 1C,D). The binding was effectively competed out by excess unlabeled wild‐type probes, but not by mutated versions (Figure 1D). Overall, these findings demonstrate that FgSge1 specifically interacts with the cis‐elements in the promoter regions of its target genes.

### 2.3 FgSge1 Binds the cis‐element of its Own Promoter for Self‐Activation

**FIGURE 2:**
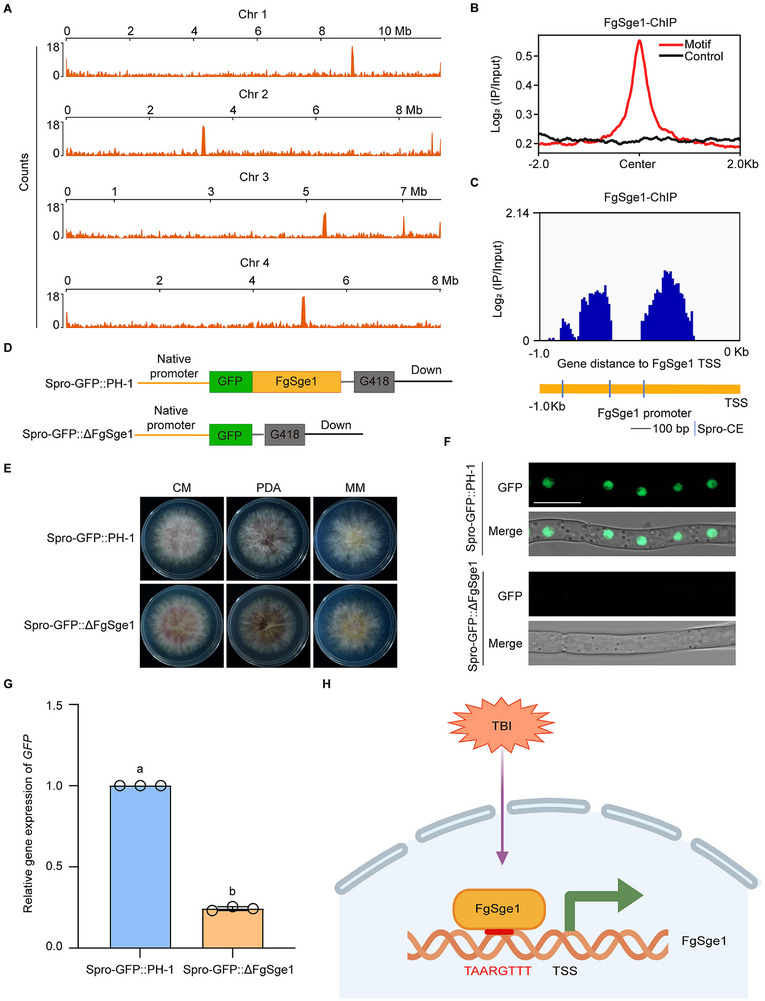
FgSge1 activates its own transcription by binding to specific motifs in its own promoter. (A) Genome‐wide distribution of the TAARGTTT motif in *F. graminearum*. (B) Metaplot of FgSge1 enrichment on TAARGTTT motif. Two biological replicates of ChIP‐seq were merged. (C) The IGV genome browser displays the enrichment of FgSge1 at its own promoter in the ChIP‐seq assay (upper panel). The cis‐elements in the FgSge1 promoter (Spro‐CE) (lower panel). (D) A schematic diagram of GFP expression driven by the FgSge1 promoter in PH‐1 (Spro‐GFP::PH‐1) and ΔFgSge1 mutant (Spro‐GFP::ΔFgSge1) strains. (E) Colony morphology of Spro‐GFP::PH‐1, and Spro‐GFP::ΔFgSge1 on CM, PDA, MM. (F) Confocal microscopy observation on FgSge1‐GFP localization in mycelia of Spro‐GFP::PH‐1 and Spro‐GFP::ΔFgSge1 in the liquid TBI medium. Bar = 10 µm. (G) The relative transcription level of *GFP* in Spro‐GFP::PH‐1 and Spro‐GFP::ΔFgSge1 under TBI conditions. Mean and standard deviation were estimated with data from three biological replicates (marked with darkdots, *n* = 3). Different letters represent statistically significant differences according to the one‐way ANOVA test (*p* < 0.05) followed by Fisher's least significant difference (LSD) test. (H) Model of FgSge1 self‐activation in response to TBI conditions through binding its own promoter‐specific cis‐elements.

To clarify the characteristics of the cis‐regulatory elements bound by FgSge1, we analyzed the genome‐wide distribution of the TAARGTTT motif. The results indicate that this motif is widely distributed, with 4126 copies present in the genome (Figure 2A). Further analysis revealed that the binding sites of FgSge1 in the genome are highly consistent with the distribution of the TAARGTTT motif (Figure 2B). These findings demonstrate that TAARGTTT is the core DNA cis‐regulatory element through which FgSge1 exerts its transcriptional regulatory functions in vivo.

Interestingly, we discovered that FgSge1 is also significantly enriched in its own promoter region (Figure 2C). A search of the FgSge1 promoter region identified three potential cis‐regulatory element sequences (Figure 2C), indicating that FgSge1 may function as a self‐regulatory factor. To test this hypothesis, we expressed GFP driven by the FgSge1 native promoter in both the PH‐1 and ΔFgSge1 strains (Figure 2D). No significant difference in hyphal growth was observed between the two strains (Figure 2E). However, under TBI conditions, subcellular localization analysis of GFP showed that fluorescence was localized to the nucleus in the wild‐type PH‐1 strain, while nuclear localization was absent in the ΔFgSge1 mutant, and the fluorescence intensity was significantly reduced (Figure 2F). Consistent with this, RT‐qPCR experiments indicated that GFP expression driven by the FgSge1 promoter was significantly lower in the ΔFgSge1 strain (Figure 2G), demonstrating that FgSge1 can activate its own expression. Taken together, these results indicate that FgSge1 can bind to the TAARGTTT cis‐elements in its own promoter, respond to TBI induction (Figure 2H). These results suggest that FgSge1 may activate its own expression, forming a positive feedback loop.

### 2.4 FgSge1 Specifically Binds to and Regulates the Transcription of SMBCs

**FIGURE 3:**
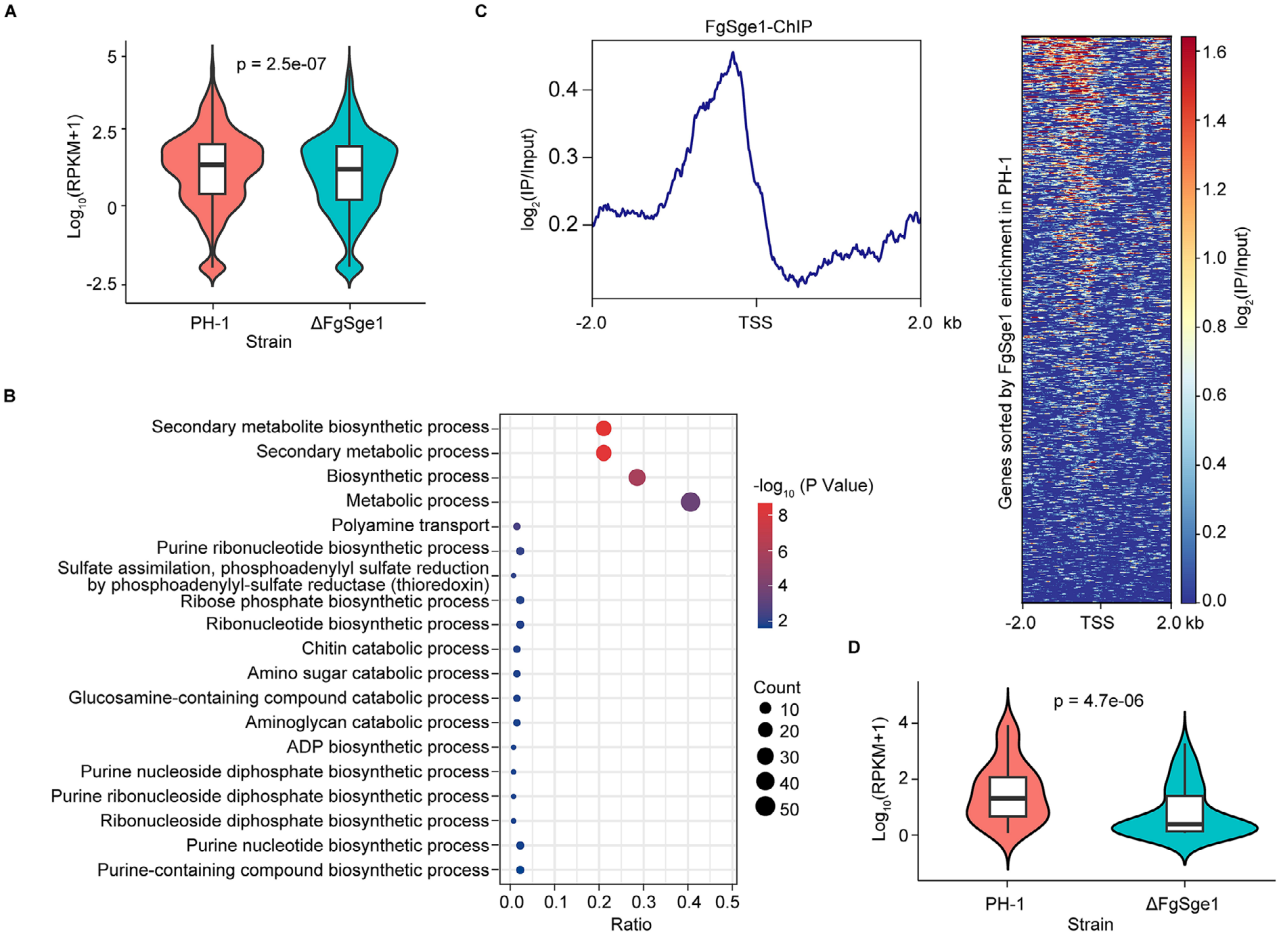
FgSge1 binds to and regulates the transcription of secondary metabolite biosynthetic genes. (A) Violin plot of gene expression (log_10_ (RPKM+1)) in PH‐1 and ΔFgSge1 at FgSge1‐binding genes. Pairwise t test was used to calculate the statistical significance. (B) GO enrichment analysis of genes with FgSge1 binding in the promoter region that are significantly down‐regulated in ΔFgSge1 transcription. Fisher's exact test was used to calculate the statistical significance. (C) Metaplot (left panel) and heatmap (right panel) of FgSge1 enrichment on SMBCs. (D) Violin plot of expression levels (log_10_ (RPKM+1)) of secondary metabolism biosynthesis gene cluster (SMBC) in the mutant and PH‐1. Pairwise t test was used to calculate the statistical significance. RPKM = Reads Per Kilobase per Milion mapped reads.

To explore the relationship between FgSge1 binding sites and gene regulation, RNA‐seq was performed on ΔFgSge1 under TBI conditions. Out of the 823 genes with FgSge1 binding sites in their promoters (Figure 1B), expression levels were significantly reduced in the ΔFgSge1 mutant (Figure 3A), indicating that FgSge1 primary acts as a positive regulator of gene transcription. Gene ontology (GO) analysis of these downregulated genes revealed enrichment in processes related to secondary metabolic process (Figure 3B). Notably, FgSge1 exhibited strong enrichment at promoter regions within SMBCs (Figure 3C). Correspondingly, transcript levels of SMBC genes were markedly decreased in ΔFgSge1 compared to the wild‐type PH‐1 (Figure 3D). Collectively, our results suggest that FgSge1 binds to promoter regions to activate transcription, particularly of secondary metabolic gene clusters, under TBI conditions.

### 2.5 FgSge1 Coordinates with the SAGA Complex to SMBCs Transcription

**FIGURE 4:**
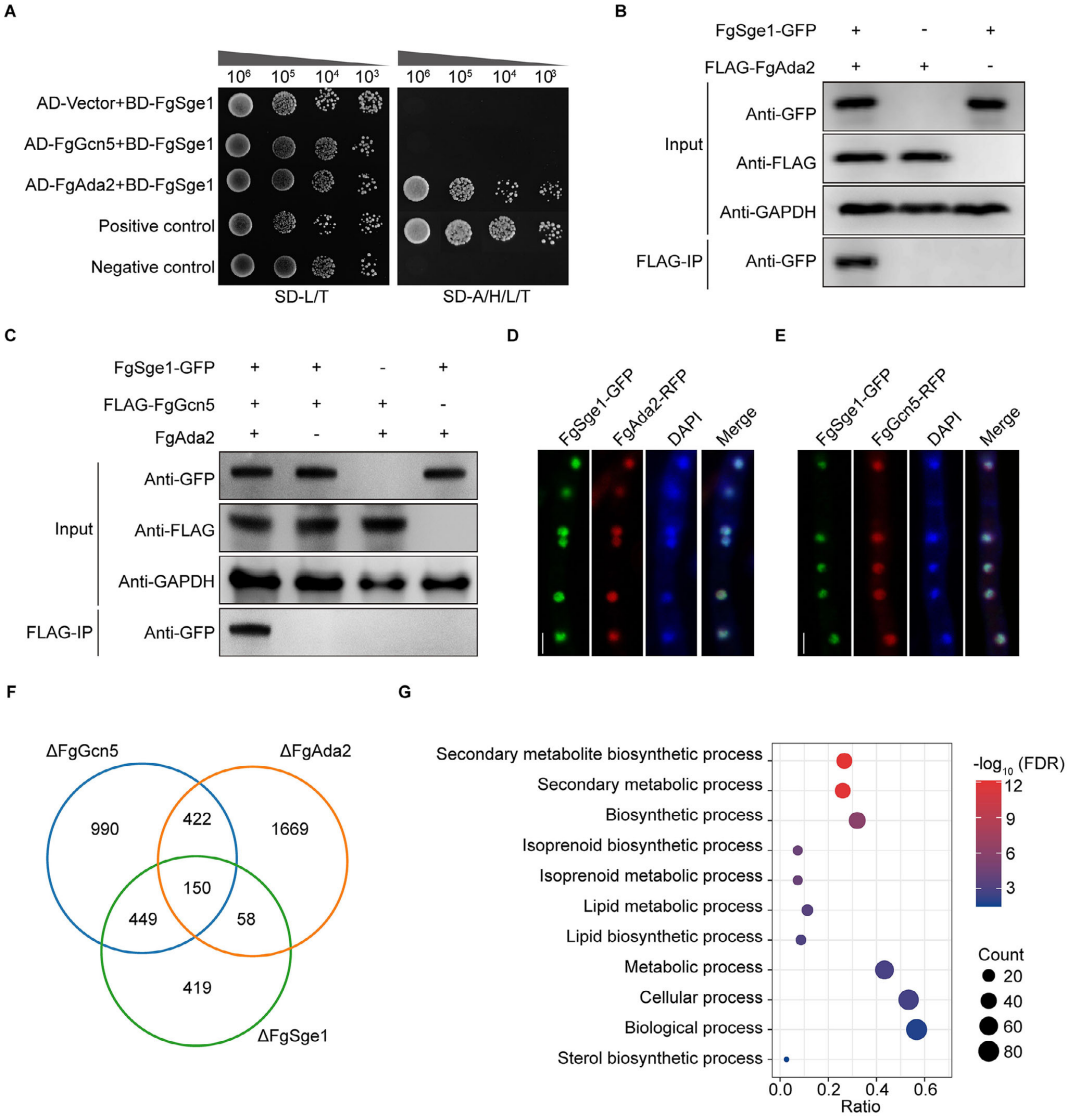
FgSge1 interacts with SAGA complex and co‐regulate the expression of secondary metabolism genes. (A) FgSge1 interacted with FgAda2 in yeast two‐hybrid (Y2H) assay. Yeast cells grew on minimal synthetic defined (SD) medium lacking leucine (L), tryptophan(T) (SD‐L/T) and medium lacking leucine (L), tryptophan(T), histidine (H), and adenine (A) (SD‐L/T/H/A). (B‐C) FgSge1 interacted with FgAda2 and FgGcn5 in the coimmunoprecipitation (Co‐IP) assay. Protein samples were extracted from the fresh mycelia growing in TBI medium. (D,E) FgSge1 and FgAda2 or FgGcn5 co‐localized into nucleus. The PH‐1 background strain containing FgSge1‐GFP and RFP tagged FgGcn5 or FgAda2 constructs was cultured in TBI for 24 h. Nuclei were labeled with the DAPI dye. Fluorescence was examined under confocal microscopy. Bars: 10 µm. (F) RNA‐seq analysis showed the overlap of significantly downregulated genes in ΔFgSge1, ΔFgGcn5, and ΔFgAda2. (G) Perform GO enrichment analysis on genes showing significant downregulation in transcription where three mutations overlapped in (F) False Discovery Rate (FDR) was used to calculate the statistical significance.

To understand how FgSge1 regulates the expression of SMBC genes, we first performed a yeast two‐hybrid screen and found that FgSge1 interact with FgAda2 directly (Figure 4A). Ada2 is a core adaptor subunit of the Spt‐Ada‐Gcn5‐acetyltransferase (SAGA) complex, working alongside Gcn5 to modulate gene expression by altering histones acetylation [19]. Furthermore, FgAda2 has been shown to form a direct physical complex with FgGcn5 in *F. graminearum* [20]. However, FgGcn5 did not show interaction with FgSge1 in the Y2H assay (Figure 4A). To further validate these interactions, we conducted Co‐IP and found the FgSge1 association with FgGcn5 and FgAda2 (Figure 4B,C). Notably, in the ΔFgAda2 strain, the association between FgSge1 and FgGcn5 was disrupted (Figure 4C). Moreover, fluorescence microscopy revealed that FgSge1 co‐localized within the nucleus with both FgGcn5 and FgAda2 (Figure 4D,E). These results collectively suggest that FgSge1 associates with the SAGA complex via directly binding to the adaptor subunit FgAda2 in *F. graminearum*. To determine whether the interaction between FgSge1 and FgAda2 depends on the N‐terminal Gti1/Pac2 domain of FgSge1, we constructed N‐ and C‐terminal truncation vectors of FgSge1 and performed Y2H assays with FgAda2 (Figure S4). The results showed that both N‐ and C‐ terminal truncations of FgSge1 interacted with FgAda2 in Y2H assays (Figure S4), suggesting that the interaction between FgSge1 and FgAda2 is likely mediated by multiple interfaces.

To investigate the regulatory mechanism, we further analyzed RNA‐seq for the ΔFgSge1, ΔFgAda2, and ΔFgGcn5 mutants under TBI conditions. A Venn diagram showed that 150 genes were commonly downregulated across all three mutants (Figure 4F). GO analysis of these common downregulated genes highlighted processes related to secondary metabolic process (Figure 4G). These results indicate that FgSge1 and the SAGA complex coordinately activate expression of secondary metabolism genes.

### 2.6 FgSge1 Regulates Transcription of Target Genes by Modulating Histone Acetylation

**FIGURE 5:**
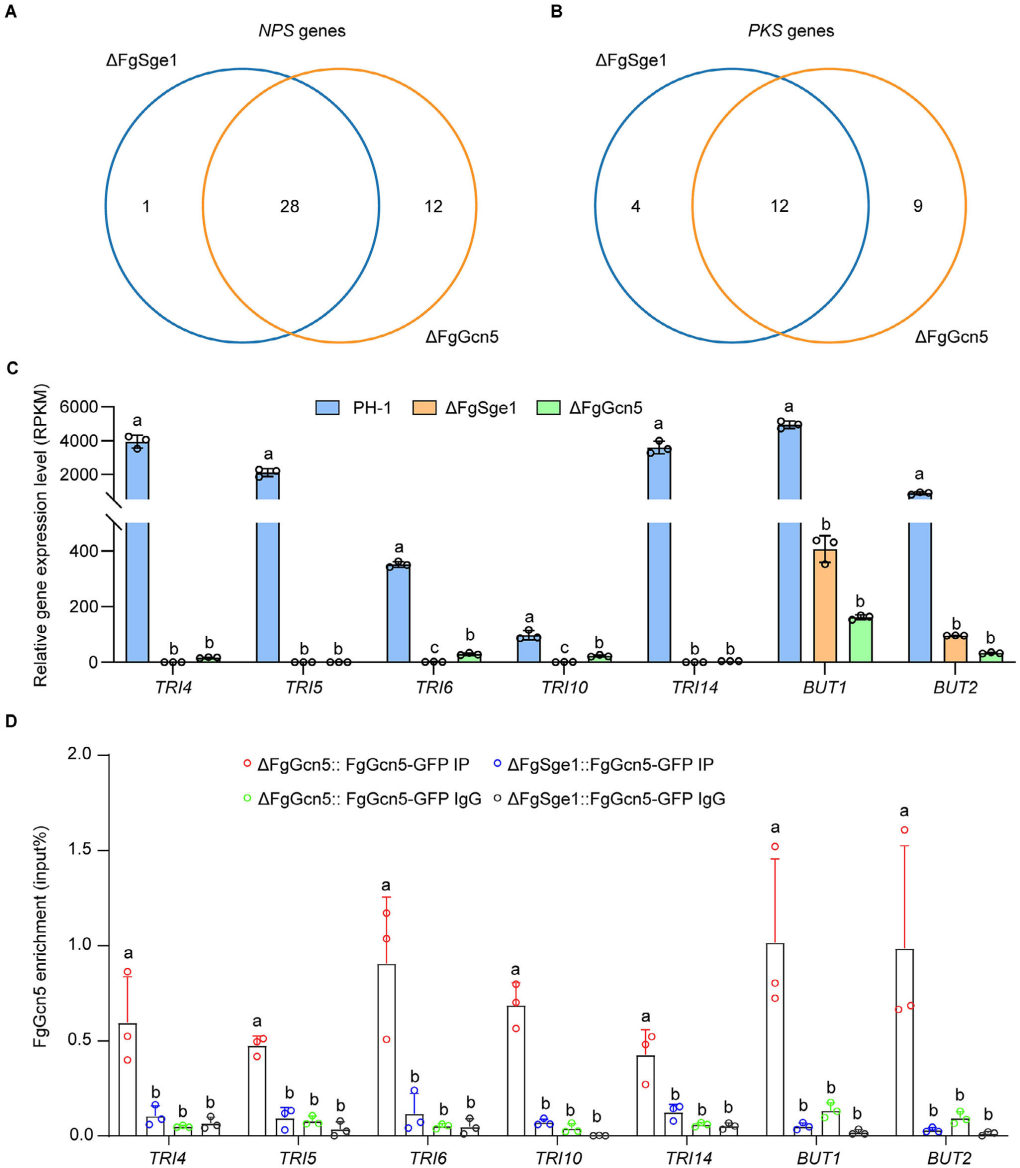
FgSge1 recruits the SAGA complex to the promoters of the target SMBC genes and regulates the transcription of these genes. (A,B) RNA‐seq analysis showed the overlap of significantly downregulated *NPS* and *PKS* genes in ΔFgSge1, ΔFgGcn5. (C) Transcript levels of *TRI*s and *BUT*s in PH‐1, ΔFgSge1, and ΔFgGcn5 analyzed by RNA‐seq. The gene expression status calculated in three biological replicates is presented. Different letters represent statistically significant differences according to the one‐way ANOVA test (*p* < 0.05) followed by Fisher's least significant difference (LSD) test. RPKM = Reads Per Kilobase per Milion mapped reads. (D) Comparison in enrichment of FgGcn5‐GFP on the promoter of SMBCs genes in TBI condition in the ChIP‐qPCR assay. Line bar in each column denotes standard deviation of three repeated experiments. Different letters indicate a significant difference (*p* < 0.05) based on one‐way analysis of variance (ANOVA) followed by Fisher's least significant difference (LSD) test for each gene.

Previous studies have indicated that FgSge1 plays a role in regulating the expression of certain *PKS* (Polyketide synthase) and *NPS* (Nonribosomal Peptide Synthetase) genes involved in secondary metabolism [6]. To comprehensively investigate whether the regulation of *PKS* and *NPS* genes mediated by FgSge1 is associated with FgGcn5, we performed a comparative analysis of significantly downregulated genes, considering the transcriptional activation role of FgSge1 (Figure 3A). Under TBI conditions, a total of 29 *NPS* genes were transcriptionally downregulated in ΔFgSge1, of which 28 were also downregulated in ΔFgGcn5 (Figure 5A). Among *PKS* genes, 16 were downregulated in ΔFgSge1, with 12 of those overlapping with the ΔFgGcn5 (Figure 5B). Notably, approximately 88.9% of the *PKS* and *NPS* genes regulated by FgSge1 were also regulated by Gcn5 (Figure 5A,B), suggesting an extensive functional overlap and coordination between FgSge1 and FgGcn5 in the regulation of these genes. In contrast, FgGcn5, which acts as an acetyltransferase, affected a larger number of *PKS* and *NPS* genes overall (Figure 5A,B).

Notably, FgSge1 regulates *TRI* and *BUT* genes, which encode well‐characterized mycotoxin secondary metabolites DON and butenolide, respectively [6]. Consistent with this, the transcription levels of these genes were markedly reduced in the ΔFgSge1 mutant (Figure 5C), and FgGcn5 is required for these genes expression (Figure 5C). Given that the SAGA is required for gene activation and secondary metabolism production in *F. graminearum* [5, 17] and FgSge1 associates with SAGA compelx (Figure 4A–E), we hypothesized that FgSge1 might recruit the SAGA complex to target gene promoters for gene expression. To test this, we performed FgGcn5 ChIP‐qPCR experiments in wild‐type and ΔFgSge1 mutant. Our results showed that FgGcn5 was enriched at the promoters of *TRI* and *BUT* genes, with this enrichment significantly reduced in ΔFgSge1 mutant (Figure 5D). Together, these findings indicate that FgSge1 binds to the promoters of SMBC genes and recruits the SAGA complex through FgAda2, thereby facilitating FgGcn5‐mediated gene transcription activation under TBI conditions.

**FIGURE 6:**
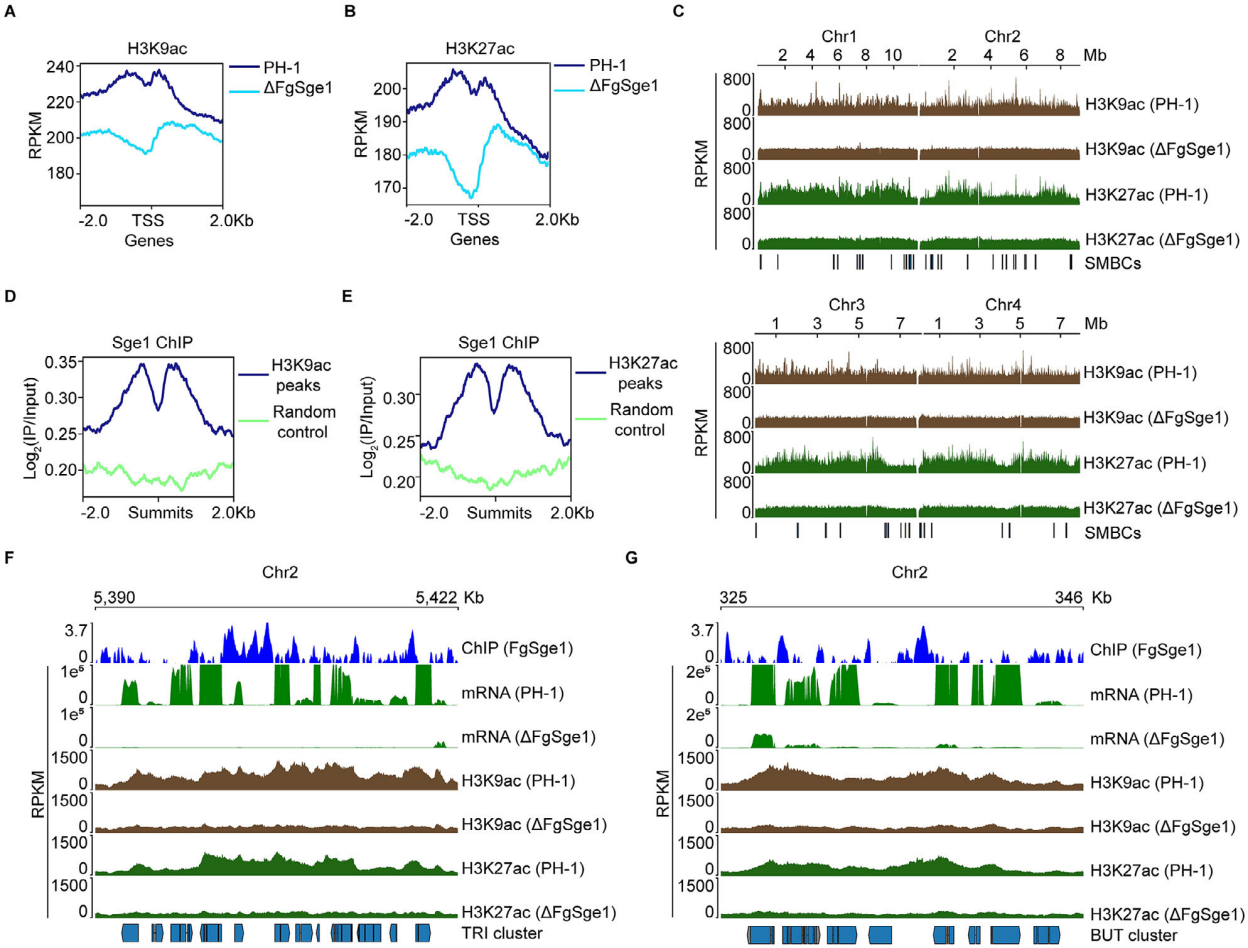
FgSge1 regulates the acetylation enrichment level of SMBCs. (A,B) Metaplot of H3K9 and H3K27 acetylation enrichment at TSS of SMBC genes in PH‐1 and ΔFgSge1. The status of H3K9ac and H3K27ac were calculated from two biological replicates. RPKM = Reads Per Kilobase per Milion mapped reads. (C) Profiles of H3K9ac and H3K27ac on genomic regions of four chromosomes in ΔFgSge1, ΔFgGcn5 and PH‐1. The status of histone modifications was calculated from two biological replicates. (D,E) Metaplot of FgSge1 binding profile at H3K9 and H3K27 acetylation peaks in PH‐1. Summits is the highest peaks of H3K9ac (D) or H3K27ac (E). The status of ChIP‐seq in WT was calculated from two biological replicates. An equal number of randomly selected regions without H3K9 and H3K27 acetylation peaks were used as a control. (F) The status of FgSge1 ChIP‐seq in WT, gene expression and histone modifications (H3K9ac and H3K27ac) of *TRI* cluster in ΔFgSge1 or WT. Two biological replicates were merged, with the Y‐axis representing Log_2_(IP/Input). (G) The status of FgSge1 ChIP‐seq in WT, gene expression and histone modifications (H3K9ac and H3K27ac) of *BUT* cluster in ΔFgSge1 or WT. Two biological replicates were merged, with the Y‐axis representing Log_2_(IP/Input).

The SAGA complex is responsible for histones acetylation. We thus assessed the H3K9ac and H3K27ac levels using Cut&Tag assays in PH‐1 and ΔFgSge1 mutants. Both modifications were notably enriched at SMBC promoters in PH‐1 but were significantly reduced in the ΔFgSge1 mutant (Figure 6A,B). Notably, the enrichment of H3K9 and H3K27 acetylation across all chromosomes was globally reduced in ΔFgSge1 mutant (Figure 6C), indicating that FgSge1 have a broader regulatory effect beyond promoters. Moreover, FgSge1 itself was enriched at sites corresponding to H3K9ac and H3K27ac marks (Figure 6D,E), suggesting a close association between FgSge1 and histone acetylation.

To further analyze the regulation of SBMCs by FgSge1, we examined the *TRI* and *BUT* gene clusters and found that FgSge1 binds to the promoter regions of *TRI* genes (including *TRI4*, *TRI5*, *TRI6*, *TRI10*, and *TRI14*) and *BUT* genes, with enrichment of H3K9ac and H3K27ac (Figure 6F,G). In the ΔFgSge1 mutant, levels of these histone marks decreased sharply (Figure 6F,G), correlating with lower genes expression and metabolism output (Figure 6F,G; Figure S2B). These findings suggest that by binding to SMBC promoters, FgSge1 modulates histone acetylation levels through SAGA complex, thereby facilitating transcriptional activation of secondary metabolic genes.

### 2.7 FgSge1 and SAGA Complex Cooperatively Regulate Chromatin Conformation

Histone acetylation in *F. graminearum* has been demonstrated to promote the formation of 3D genome structures at secondary metabolic gene clusters, with these chromatin loops, called jet‐like domains, positively correlating with active gene transcription [18]. Since FgSge1 can recruit SAGA complex and ΔFgSge1 mutant exhibited global downregulation of histone acetylation (Figures 5 and 6), we thus performed High‐throughput chromosome conformation capture (Hi‐C) analysis comparing the PH‐1 and ΔFgSge1 strains, using two biological replicates to ensure data quality consistency (Figure S5). The analysis revealed a global alteration in chromosome conformation in ΔFgSge1 compared to PH‐1 (Figure S5A). The long‐distance inter‐ and intra‐chromosomal interactions were significantly reduced in the mutant, as indicated by a general decreased off‐diagonal signals in the interaction map (Figure S5A). By dividing the ΔFgSge1 by the PH‐1 data, it is apparent that long‐distance interactions decrease globally both inter and intra‐chromosomally in ΔFgSge1, as shown by general blue signals far from the diagonal line (Figure S5A). However, local intra‐chromosomal interactions represented by dense red signals along the diagonal line are favored in ΔFgSge1. The distance decay of interactions shows interactions below 700 kb are enriched in ΔFgSge1 vs. PH‐1 (Figure S5B). The 3D modeling analysis of the genomic chromatin architecture in PH‐1 and ΔFgSge1 (Figure S5C) revealed that, at equivalent scales, distal chromatin in ΔFgSge1 exhibited greater relaxation compared to PH‐1, indicating significant alterations in the global chromatin conformation. These findings suggest that FgSge1 plays a critical role in regulating the 3D conformation of chromatin in *F. graminearum*.

**FIGURE 7:**
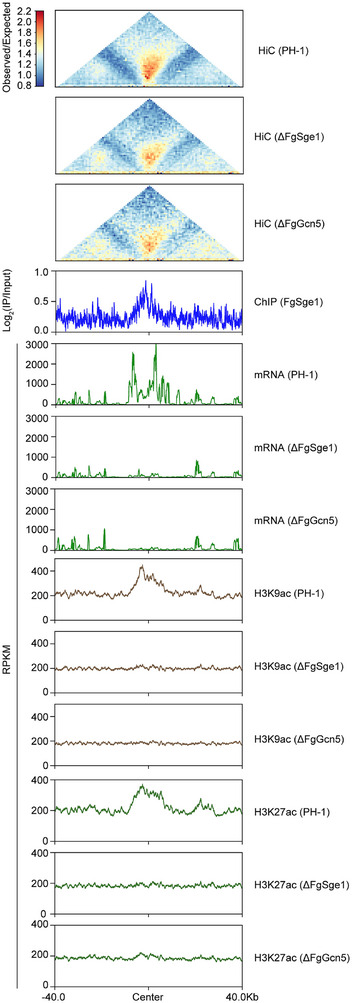
FgSge1 and FgGcn5 cooperatively regulate chromatin jet‐like domains to coordinate gene expression. Characterization of the down‐regulated jet‐like domains (*n* = 23) identified in the ΔFgSge1 mutant. All jet‐like domains and their 40‐kb upstream and downstream flanking sequences were aggregated for integrated analysis to visualize their Hi‐C interaction patterns, FgSge1 binding signals, gene expression levels, and H3K9ac and H3K27ac modification profiles. Observed‐over‐expected matrices (1 kb resolution) of the 23 selected jet‐like domains in ΔFgSge1, ΔFgGcn5, or the wild‐type strain PH‐1 are presented.

Jet‐like chromatin structures, spanning approximately 54 kb, have been shown to be associated with rapid gene activation [18]. We therefore quantitatively examined these specialized chromatin domains in the ΔFgSge1 mutant, focusing on jet‐like domains that were weakened. In total, 23 jet‐like domains exhibited a significant reduction in strength in ΔFgSge1 (Figure 7). Given the established link between histone acetylation and jet‐like domain formation [18], we next analyzed changes in the strength of these 23 jet‐like domains in the ΔFgGcn5 mutant (Figure 7). Notably, all 23 domains showed similarly decreased strength in both ΔFgGcn5 and ΔFgSge1 (Figure 7). Integrated analysis of these jet‐like domains together with their upstream and downstream flanking regions revealed significant enrichment of FgSge1 at these domains. Using H3K9ac and H3K27ac as markers of FgGcn5‐mediated histone acetylation, we observed that both marks were highly enriched at these jet‐like domains in the wild‐type strain PH‐1 (Figure 7). In contrast, deletion of either FgSge1 or FgGcn5 resulted in a pronounced reduction in H3K9ac and H3K27ac levels, accompanied by significantly decreased transcription of genes within these 23 jet‐like regions (Figure 7). Collectively, these results indicate that FgSge1 cooperates with FgGcn5 to regulate histone acetylation, maintain the strength of jet‐like chromatin domains, and promote transcriptional activation of genes within these domains.

**FIGURE 8:**
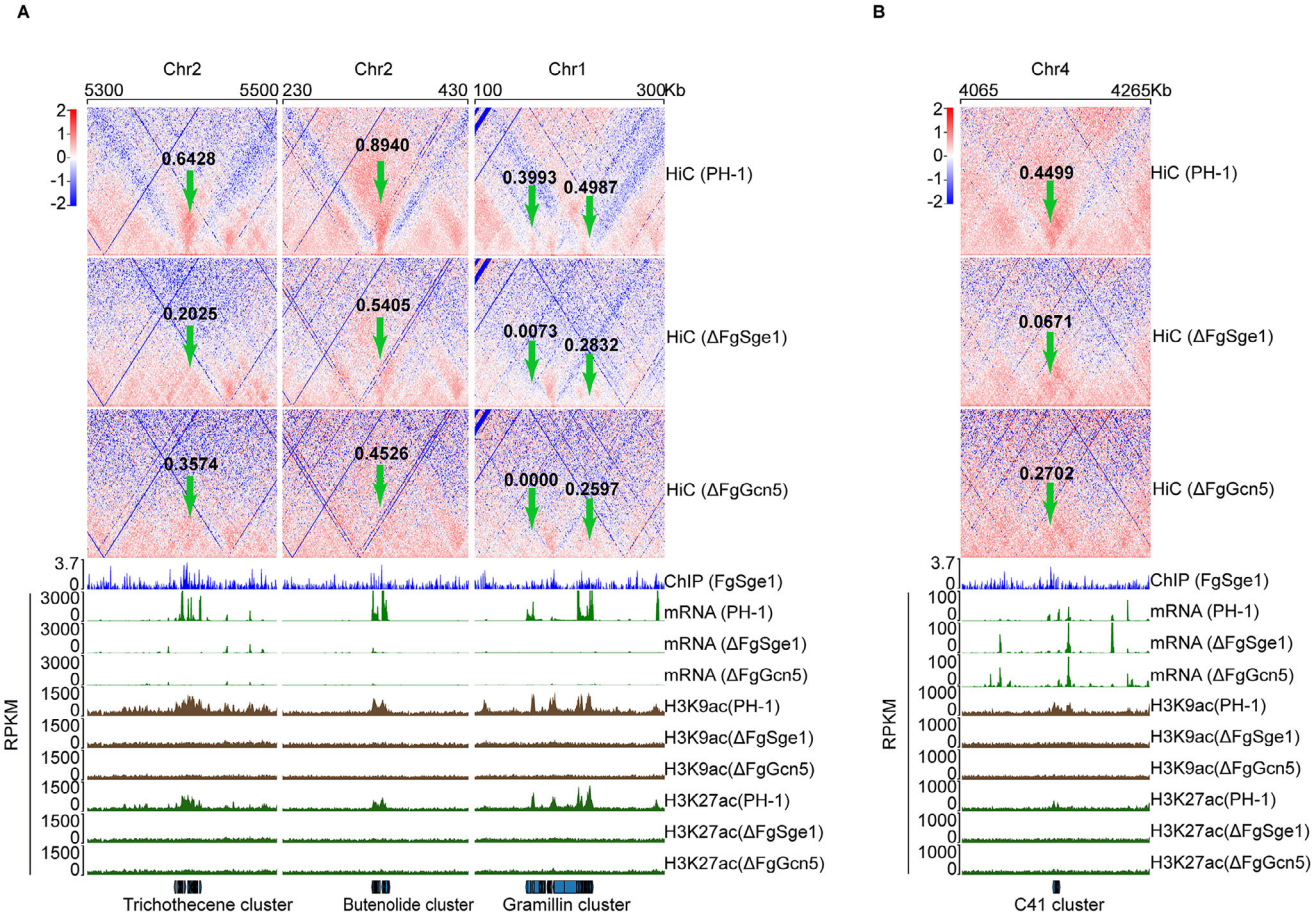
FgSge1 regulates histone acetylation to modulate jet‐like structures at SMBCs. (A) Observed over expected matrices (1 kb resolution) of selected SMBCs in ΔFgSge1, ΔFgGcn5, or PH‐1. Numbers indicate the strength of jet‐like domain. The Hi‐C data are two biological replicates of PH‐1 and one biological replicate of mutants were combined to calculate the values. Two biological replicates of ChIP‐seq, with the Y‐axis representing Log_2_(IP/Input), three biological replicates of RNA‐seq, and two biological replicates of Cut&tag were merged. (B) Observed over expected matrices (1 kb resolution) of selected SMBCs of unknown products (C41) in ΔFgSge1, ΔFgGcn5, or PH‐1. Numbers indicate the strength of jet‐like domain. The Hi‐C data are two biological replicates of PH‐1 and one biological replicate of mutants were combined to calculate the values. Two biological replicates of ChIP‐seq, with the Y‐axis representing Log_2_(IP/Input), three biological replicates of RNA‐seq, and two biological replicates of Cut&tag were merged.

Importantly, among the 23 significantly down‐regulated jet‐like domains, 6 belong to SMBCs. This is consistent with previous reports that jet‐like domains are essential for the rapid transcription of SMBCs [18]. A decrease in their strength indicates weaker jet‐like structures. Jet‐like domains on SMBCs, including *TRI*, *BUT*, and *GRA* (gramillin) clusters, were significantly weaker in both ΔFgSge1 and ΔFgGcn5 compared to the wild‐type PH‐1. Specifically, the jet strength around the *TRI* cluster decreased from 0.6428 in PH‐1 to 0.2025 in ΔFgSge1 and 0.3574 in ΔFgGcn5 (Figure 8A). For the *BUT* cluster, the strength dropped from 0.8940 in PH‐1 to 0.5405 in ΔFgSge1 and 0.4526 in ΔFgGcn5 (Figure 8A). The *GRA* cluster showed even more dramatic reductions: one jet was nearly gone in ΔFgSge1, and completely absent in ΔFgGcn5 (strength = 0) (Figure 8A). Another jet domain with a strength of 0.4987 in PH‐1 was reduced to 0.2832 and 0.2597 in ΔFgSge1 and ΔFgGcn5, respectively (Figure 8A). Additionally, other SMBCs, like the *C41* cluster, also showed decreased jet strength in the mutants (Figure 8B). Overall, global jet strength across SMBCs was lower in both ΔFgSge1 and ΔFgGcn5 mutants, which matched the decreased gene expression of these genes (Figure 8A,B). The results suggest that FgSge1 is required for maintain histone acetylation and the formation of jet structures at SMBCs during TBI conditions, which is important for their activation.

## 3 Discussion

**FIGURE 9:**
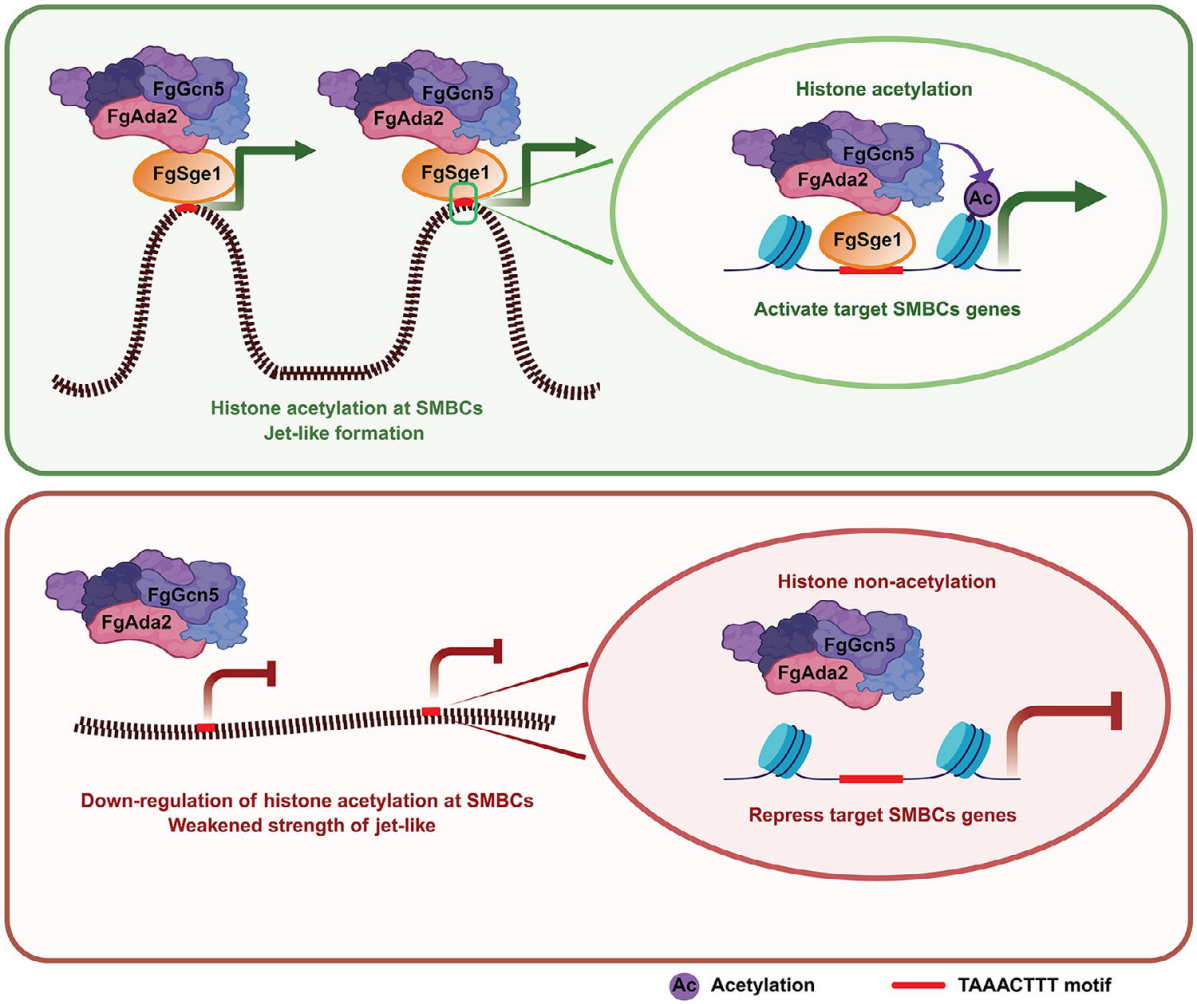
The molecular mechanism model of FgSge1 regulation of SMBC gene transcription under toxin induction conditions in *F. graminearum*. FgSge1 recruits the SAGA complex to specific cis‐elements for histone acetylation of SMBCs, which is essential for jet‐like at SMBCs. SMBCs form jet‐like structures, and gene transcription is activated in rapid response to TBI‐inducing conditions (upper panel). In the absence of FgSge1, histone acetylation of SMBCs genes is significantly reduced. The chromatin jet‐like structures are diminished, and gene transcription is repressed (lower panel).

Our study uncovers a novel epigenetic regulatory mechanism in *F. graminearum*, demonstrating that the transcription factor FgSge1 functions as a critical hub, linking transcription factor activity with epigenetic modifications and 3D genome organization. FgSge1 directly binds to cis‐elements within the promoters of SMBCs and activates their transcription. This activation is mediated through the recruitment of the SAGA complex via FgAda2, promoting histone acetylation at H3K9 and H3K27, which correlates with local chromatin architecture and increased gene expression. Importantly, FgSge1 also orchestrates higher‐order chromatin architecture, as indicated by Hi‐C analyses showing that deletion of FgSge1 leads to widespread disruptions in chromosome conformation and weakened jet‐like chromatin domains associated with active SMBCs. These findings establish FgSge1 as a key regulator that integrates histone acetylation and chromatin organization to control secondary metabolism and mycotoxin production (Figure 9).

Notably, Sge1 is conserved among fungal kingdom (Figure S1), and no homologous genes have been identified in animals or plants (Figure S1). While FgSge1 is required for the virulence of numerous fungal species [6, 8, 9, 10, 21, 22], it is not exclusive to pathogenic fungi (Figure S1). This is consistent with the functional divergence of Sge1 orthologs across different species [6, 7, 10, 23]. The expression level and functional activity of FgSge1 are precisely regulated through multiple layers of control. At the transcriptional level, FgSge1 activates its own transcription by binding to its own promoter, a mechanism that rapidly amplifies its transcriptional signal and drives the expression of secondary metabolite biosynthetic clusters (SMBCs) (Figure 2). While the current evidence supports the existence of this positive feedback loop, its genetic architecture could be further dissected in future work, for instance, by introducing specific mutations into the cis‐elements to formally prove they are necessary for self‐activation. It is necessary to generate mutants that specifically impair the binding of FgSge1 to its own promoter. Our current results cannot rule out the influence of other regulatory proteins caused by the loss of FgSge1. In addition, the recruitment function of the SAGA complex in regulating FgSge1 self‐expression also remains to be elucidated. At the post‐transcriptional level, its activity and stability are dynamically modulated by post‐translational modifications, with phosphorylation plays a critical role. Previous studies have shown that the conserved T67 site in the FgSge1 protein serves as a potential protein kinase A (Pka) phosphorylation site, and mutation at this site significantly impairs FgSge1 function [8]. Therefore, the integration of transcriptional amplification and post‐translational fine‐tuning allows for the precise spatiotemporal control of FgSge1 activity, which is essential for its biology roles.

Under vegetative growth conditions, the markedly reduced enrichment of the SAGA complex at *TRI* gene loci has important biological implications [5]. This reduction reflects a fungal strategy for resource optimization during the basal metabolic phase: by maintaining low levels of FgSge1 expression and limiting SAGA complex recruitment to toxin biosynthetic genes, fungi effectively minimize unnecessary energy expenditure. As a result, chromatin at these loci remains in a relatively closed state under non‐pathogenic conditions, thereby repressing transcription of toxin biosynthesis–related genes. Importantly, this reversible enrichment pattern highlights the sophisticated adaptive capacity of fungi to respond to environmental cues. Upon exposure to inductive signals, such as growth in TBI medium or host infection, a rapid amplification cascade is triggered through FgSge1 auto‐activation and post‐translational modifications, leading to targeted recruitment of the SAGA complex. This dynamic regulatory mechanism not only explains the stage‐specific induction of toxin biosynthesis but also underscores the role of the FgSge1–SAGA axis as a signal‐responsive regulatory hub.

Although Gcn5 and the SAGA complex are required for fungal pathogenesis and importantly, phenazine‐1‐carboxamide (PCN) secreted by biological bacterium is able to directly bind and suppress its activity [1], their conservation across eukaryotes limit their potential as perfect targets for disease control. Since FgSge1 lacks direct homologs in plants (such as wheat, rice, arabidopsis) or animals (including humans and mice), targeting FgSge1 presents a promising strategy to reduce mycotoxin contamination and impede fungal virulence. Current promising approaches include Host‐Induced Gene Silencing (HIGS), where expressing double‐stranded RNA targeting FgSge1 in wheat has been shown to suppress pathogen toxin‐related genes, significantly decreasing disease severity and mycotoxin levels [24]. Additionally, exploring small‐molecule inhibitors that interfere with Sge1's nuclear localization or DNA‐binding capacity—such as compounds targeting its conserved nuclear localization signal—represents an important avenue for future research.

The SAGA complex, particularly its Gcn5 subunit, is well‐established as a critical epigenetic modifier involved in gene activation through histone acetylation. In various systems, TFs are known to recruit Gcn5 and the SAGA complex to specific target genes, thereby facilitating localized chromatin modifications that promote transcription, such as *Arabidopsis* bZIP11 and wheat CAMTA2, guiding GCN5‐mediated acetylation and activation of specific genes [15, 25]. Our findings extend this paradigm by demonstrating that in *F. graminearum*, FgSge1 interacts with FgAda2 to recruit the Gcn5‐containing SAGA complex specifically to SMBCs, promoting localized histone modifications and mycotoxins production. However, different TFs can exert distinct biological effects upon interacting with the SAGA complex. For instance, in *F. graminearum*, the APSES‐type TF FgStuA activates *TRI6* expression via SAGA recruitment and subsequent acetylation [5], whereas the TF FgPacC30 functions as a repressor by inhibiting the acetyltransferase activity of SAGA to downregulate iron metabolism genes [20]. These contrasting roles highlight the diverse outcomes of SAGA engagement depending on the specific TF involved. Additionally, it is intriguing to investigate how different TFs coordinate to regulate the expression of genes involved in the same process. It is highly likely that a regulatory network exists in the modulation of SMBCs by FgSge1 and other transcription factors. Whether FgSge1 and these transcription factors exhibit a temporal order, competitive interactions, or antagonistic effects when regulating the same target genes (e.g., *TRI*s), as well as the specific underlying mechanisms, remains to be further elucidated. Notably, the SAGA complex exerts broader influence on chromatin organization, affecting the formation of jet‐like domains and global gene expression regulation [18]. Our data extend the role of FgSge1 beyond local chromatin modification to the orchestration of large‐scale chromatin architecture, which may be vital for rapid and efficient gene activation during toxin biosynthesis and virulence. Whether additional SMBC‐regulating TFs participate in shaping chromatin conformation, and how their activities intersect with FgSge1‐mediated regulation, represents an important avenue for future investigation.

In *F. graminearum*, jet‐like chromatin structures display lower interaction strength than classical chromatin loops [18]. In mammalian cells, the establishment of topologically associating domains (TADs) largely relies on cohesin‐mediated loop extrusion [26, 27]; however, whether a similar mechanism operates in *F. graminearum* remains unknown. During chromatin loop formation, histone acetylation reduces the rigidity of chromatin fibers by neutralizing the positive charges of lysine residues in histone tails [19], thereby facilitating loop extrusion by cohesin. In addition to its biophysical effects, histone acetylation contributes functionally to TAD boundary establishment through CTCF‐dependent mechanisms [28] (. Notably, histone acetylation marks such as H3K9ac and H3K27ac are biochemically and genetically linked to the recruitment of cohesin loaders, including NIPBL, often mediated by BET family proteins such as BRD4, thus directly influencing cohesin loading and 3D genome organization [29]. In *F. graminearum*, FgSge1 recruits the SAGA complex, which is essential for the formation of jet‐like chromatin structures by regulating local histone acetylation levels. Through modulation of histone acetylation, this process likely alters chromatin conformation and may involve cohesin or other epigenetic regulators. Elucidating this fungus‐specific chromatin regulatory mechanism will not only provide evolutionary insights into 3D genome organization but may also uncover novel targets for antifungal intervention.

In addition to its well‐characterized role in histone acetylation, recent studies have highlighted the capacity of Gcn5 and the SAGA complex to acetylate a variety of non‐histone substrates, adding an important layer of functional diversity to their regulatory capabilities. For instance, in yeast and mammalian systems, Gcn5 can directly modify its co‐subunit Ada3, enhancing the enzymatic activity of the complex and facilitating cellular adaptation to stress [30]. In plants, Gcn5 similarly mediates multi‐site acetylation of the co‐factor ADA2, which influences its stability and degradation via ubiquitination pathways [31]. Importantly, in *F. graminearum*, the SAGA complex has been found to acetylate non‐histone proteins such as FgAtg8 at the K13 site, a modification critical for regulating autophagy and, consequently, DON biosynthesis [32]. Our findings suggest that the SAGA complex's functions extend beyond chromatin modification to include direct non‐histone protein acetylation, which may further regulate metabolic and stress response pathways. This dual role of SAGA reveals a sophisticated regulatory system where non‐histone acetylation participates in integrating metabolic signals, environmental cues, and gene expression, offering new insights into fungal epigenetic control and potential targets for mitigating virulence and toxin production.

## 4 Materials and Methods

### 4.1 Fungal Strain Construction

The wild‐type (WT) strain PH‐1 (NRRL 31084) of *F. graminearum* was used as a parental strain for transformation experiments. Construction of gene deletion mutants using polyethylene glycol (PEG)‐mediated protoplast transformation method as previously described [33]. Briefly, PH‐1 fresh mycelia were treated with driselase (D9515, Sigma, MO, USA), lysozyme (RM1027, RYON, Shanghai, China), and cellulose (RM1030, RYON, Shanghai, China) to obtain protoplasts. Fusion DNA fragments for target gene deletions were constructed using the double‐joint (DJ) PCR method [34]. Primers used to amplify the sequences flanking each gene are listed in Table S1. The open reading frame (ORF) of each gene was replaced with the hygromycin resistance (HPH) gene or nourseothricin resistance gene, and putative gene deletion mutants were confirmed by PCR assay. For complementation, ORFs of the corresponding genes were fused to the geneticin (G418) resistant gene and a fluorescent protein gene (e.g., GFP), and the fused DNA fragments were introduced into the corresponding deletion mutants. For the construction of GFP‐tagged strains driven by the native FgSge1 promoter, the upstream and downstream flanking fragments of the FgSge1 promoter and ORF, the FgSge1 promoter fragment, the GFP fragment, and the resistance cassette were amplified using the primers listed in Table S1. Following fragment fusion and ligation, the resulting construct was separately transformed into the wild‐type strain PH‐1 and the ΔFgSge1 mutant.

### 4.2 Fungal Culture Conditions

For mycelial growth assays, each strain was grown in complete medium (CM) (10 g glucose, 2 g peptone, 1 g yeast extract, 1 g casein amino acids, 6 g NaNO_3_, 0.52 g KCl, 0.52 g MgSO_4_·7 H_2_O, 1.52 g KH_2_PO_4_, 1 mL trace elements, 0.01% vitamins, 10 g agar, and 1 L water, pH 6.5), minimal medium (MM) (10 mm K_2_HPO_4_, 10 mm KH_2_PO_4_, 4 mm (NH_4_)_2_SO_4_, 2.5 mm NaCl, 2 mm MgSO_4_, 0.45 mm CaCl_2_, 9 mm FeSO_4_, 10 mm dextrose, 1% agar, and 1 L water, pH 6.9), or potato dextrose agar (PDA) (200 g potato, 20 g glucose, 10 g agar, and 1 L water). For the pigment assay, each strain was incubated in potato dextrose water (PDB) (200 g potato, 20 g glucose, and 1 L water). Toxin biosynthesis inducing (TBI) medium (30 g sucrose, 1 g KH_2_PO_4_, MgSO_4_.7H_2_O, 0.5 g KCl, 0.01 g FeSO_4_.7H_2_O, 1.5 g putrescine, and 1 L water, pH 4.5) was used to culture each strain for DON measurement and fluorescence observation.

### 4.3 Virulence and DON Production Assay

Virulence measurements on flowering wheat heads, wheat germ sheaths, and maize whiskers were performed as described previously [35, 36]. The strains were cultured on PDA plates at 25°C for 3 days to obtain fresh mycelia. A flower spikelet of each wheat head (the susceptible variety Jimai 22) was inoculated with fresh mycelia of each strain, and the inoculated wheat plants were incubated at 22 ± 2°C and 95%–100% humidity after inoculation. The symptoms were examined and photographed 15 d after inoculation. In addition, the germ sheath of wheat and maize silks were inoculated with fresh mycelial plugs and incubated at 25°C and 95%–100% humidity, and the symptoms were observed and photographed 7 d after inoculation. There were 20 replicates for each strain, and the experiment was repeated three times.

To determine the production of DON, each tested strain was incubated in TBI at 28°C for 7 days at 150 rpm. The supernatant was collected for DON extraction, and the mycelia were dried and weighed. The DON production of each sample was quantified by the DON Quantification Kit Wis008 (Weisai, China). The experiment was repeated three times.

### 4.4 Phenotype Determination

To analyze penetration ability, fresh mycelium discs were inoculated onto the surface of cellophane membranes placed on MM medium. After incubation at 25°C for 2 days, the cellophane membranes were removed. Following a further 2 days of incubation, mycelium growth on each culture plate was observed. Mycelium growth on the culture plates indicated that the mycelium had penetrated the cellophane membranes. This experiment was repeated three times.

To determine the response of mycelium to cell wall stress, sodium dodecyl sulfate (SDS) and Congo red (CR) were used as stress agents. Mycelium plugs from each strain were inoculated onto CM agar plates supplemented with 0.02% SDS or CR. After incubation at 25°C in the dark for 2 days, mycelial growth on each plate was observed. This experiment was repeated three times.

### 4.5 Staining and Microscopic Examination

Fluorescence intensity and localization of the tagged proteins were observed under a Zeiss LSM780 confocal microscope (Niedersachsen, Germany). The fluorescence intensity of protein FgSge1 was observed by incubating the complement strain in MM and TBI at 28°C with 150 rpm shaking for 36 h. To check the co‐localization of proteins FgSge1, FgGcn5, and FgAda2, each strain containing the marker protein was constructed and incubated in TBI at 28°C with 150 rpm shaking for 36 h. Cell nuclei were stained with DAPI and then examined microscopically. The parameters of the confocal microscope were set as follows: green fluorescence laser excitation at 488 nm, blue fluorescence DAPI excitation at 405 nm, red fluorescence RFP excitation at 561 nm, and fluorescence intensity was obtained using Zeiss ZEN 2010 software. The experiment was repeated three times.

### 4.6 ChIP‐seq and ChIP‐qPCR Assays

Each strain was cultured in TBI medium at 28°C for 36 h. ChIP was performed based on a published protocol with minor modifications [37]. Briefly, fresh mycelia were cross‐linked with 1% formaldehyde for 15 min and then stopped with 125 mm glycine. The resulting mycelia were ground in liquid nitrogen and resuspended in the lysis buffer (250 mm, HEPES pH 7.5, 150 mm NaCl, 1 mm EDTA, 1% Triton, 0.1% Deoxycholate, 10 mm DTT) and protease inhibitor (Sangon Biotech Co. Ltd). Subsequently, DNAs were sheared into 200–500‐bp fragments by a Bioruptor Plus UCD‐300 (Diagenode, Seraing, Belgium) with 30 s on and 30 s off. After centrifugation, the supernatant of each sample was mixed with the rabbit polyclonal anti‐GFP antibody (ab290, 1: 500 dilution; Abcam, Cambridge, UK), together with the protein A agarose beads (sc‐2001; Santa Cruz Biotechnology Co. Ltd, Santa Cruz, CA, USA). After washed orderly by low salt, high salt, LiCl wash buffers, and TE buffer, DNA was eluted from beads with the elution buffer; and then, steps of washing, eluting, reversing cross‐linking, and digesting with proteinase K were conducted sequentially for the samples. Subsequently, DNA was precipitated by phenol/chloroform/isoamyl ethanol. Finally, the ChIP‐enriched DNA samples were sequenced with an Illumina HiSeq 2500 (Genergy Biotechnology Co. Ltd, Shanghai, China) or used for quantitative PCR analysis using SYBR green I fluorescent dye detection with the corresponding primers (Table S1). Raw reads of ChIP‐seq were processed to remove adapters using Fastp (v.0.23.3) [38]. The cleaned reads were aligned to the PH‐1 genome using Bowtie2 (v.2.4.5), and the unique alignments of ChIP‐seq data were used to call peaks using MACS2 (v2.2.7). The peaks were annotated using bedtools (v2.30.0). RPKM normalized Coverage tracks with a bin size of 10 bp were calculated using the bamCoverage in Deeptools (v.3.5.0). In addition, to identify the binding cis‐element of FgSge1, 500‐bp sequences around the peak summits were extracted and used for motif identification with the Sensitive, Thorough, Rapid, Enriched Motif Elicitation (STREME) program [39]. ChIP‐Seq was independently performed twice, and overlapped results were presented. ChIP‐qPCR was independently repeated three times.

### 4.7 RNA‐seq Assay and RT‐qPCR

Each strain was cultured in TBI medium for 36 h for RNA‐seq analysis. Total RNA was extracted using the RNA Isolation Kit (Tiangen, China), and RNA samples were evaluated for quantity and completeness using the RNA Nano 6000 Analysis Kit on a Bioanalyzer 2100 system (Agilent Technologies, Santa Clara, CA, USA). Constructed libraries were sequenced in 2 × 150‐bp mode on the Illumina NovaSeq platform as previously described [18]. Cleared reads were mapped to the PH‐1 reference genome using HISAT2 (v.2.2.1) [40] (FungiDB release 58, https://fungidb.org/fungidb/app). Finally, unique comparisons were calculated using HTSeq‐count (v.0.11.3) [41] and differentially expressed genes were identified using DESeq2 (v.1.36.0) [42] under conditions of adjusted *p*‐value < 0.05 and log_2_fold change >1 or <‐1. Gene expression coverage traces for the 1‐kb genome bin were calculated using bamCoverage in the deepTools package (v3.5.0) [43], with read coverage normalized by RPKM. The experiment was repeated three times independently.

For RT‐qPCR, total RNA from each strain was isolated from fresh mycelia as described above. For genomic DNA digestion and cDNA synthesis, the HiScriptII Q RT SuperMix for qRCR kit was used. The relative expression of the target genes was measured on a Bio‐RAD Real‐Time Analyzer using a SYBR qPCR Master Mix kit (Vazyme Biotechnology). The FgACTIN gene was used as an internal control. Primer sequences for qRT‐PCR are listed in Table S1. The experiment was repeated three times independently.

### 4.8 Protein Purification and EMSA

The coding sequences of FgSge1 were amplified from cDNA, and the fragment was then inserted into pGEX‐4T‐3, to generate a construct for GST‐tagged protein. The plasmids were then transformed into the *Escherichia coli* strain BL21. The obtained GST‐tagged protein was purified with GST agarose beads (C600031; BBI, Shanghai, China) and eluted with reduced glutathione solution.

For EMSA, DNA fragments of the 3 times repeated cis‐element of FgSge1 and the mutated cis‐element were labeled with biotin. Probe DNA labeled with or without biotin at the 5' end was synthesized by Sangon Biotech Co. The FgSge1‐GST protein was purified as described above. EMSA was performed using the Chemiluminescent EMSA Kit (Beyotime Biotechnology). Briefly, a mixture of FgSge1‐GST protein and biotin‐labeled probe was incubated at room temperature for 10 min, and then the reaction was electrophoresed on a 6% polyacrylamide gel in 0.5× TBE and transferred to a positively charged nylon membrane (INYC00010; Millipore Corp.). The signal was detected using the chemiluminescent substrate in the kit according to the manufacturer's instructions. Experiments were performed three times independently.

### 4.9 Y2H Assays

To construct the plasmids for Y2H analysis, the coding sequence of each gene was amplified from the cDNA of PH‐1 using the appropriate primer pairs (Table S1). cDNA fragments were cloned into the yeast GAL4‐binding domain vector, pGBKT7, and the GAL4 activation domain vector, pGADT7 (Clontech, Mountain View, CA, USA), respectively. The Y2H plasmid pair was cotransformed into *S. cerevisiae* strain Y2H Gold according to the lithium acetate/single‐stranded DNA/polyethylene glycol transformation protocol. Plasmid pairs pGBKT7‐53 and pGADT7‐T were used as positive controls. Plasmids pGBKT7‐Lam and pGADT7‐T were used as negative controls. Transformants were grown on synthetic defined (SD) lacking Leu and Trp at 30°C for three days. Yeast cell dilutions were further transferred to SD free of His, Leu, Trp, and Ade to assess protein‐protein interactions. Three experiments were performed for each Y2H assay.

### 4.10 Co‐IP Assay

The target genes were fused to different tags (GFP or 3×FLAG), and the vectors were verified by DNA sequencing and pairwise transferred into the PH‐1 strain. The PH‐1 strain harboring FgGcn5‐FLAG and FgSge1‐GFP tags was subjected to homologous recombination as described in Section 4.1, with the nourseothricin resistance cassette used to knockout the FgAda2 gene. Western blotting was used to confirm transformants containing a pair of fusion constructs. For Co‐IP assays, fresh mycelia (700 mg) of each strain were finely ground and suspended in 1 mL of extraction buffer (50 mm Tris–HCl, pH 7.5, 100 mm NaCl, 5 mm EDTA, 1% Triton X‐100) containing 10 µL of protease inhibitor mixture (Sangon Biotech Co.Ltd, Shanghai, China). Lysates were placed on ice for 15 min and centrifuged at 12 000 *g* for 10 min at 4°C. The supernatants were incubated with the Anti‐DYKDDDDK magnetic agarose (Thermo Scientific). Western blot assays were formed using 1:5000 dilution of mouse monoclonal anti‐FLAG (A9044; Sigma) and rabbit polyclonal anti‐GFP (ab32146; Abcam, Cambridge, UK) antibodies to analyze proteins eluted from anti‐FLAG magnetic agarose. In addition, 1:5000 dilution of murine monoclonal anti‐GAPDH antibody (EM1101; HuaBio Co. Ltd., Hangzhou, China) was used as a reference. The goat polyclonal anti‐rabbit IgG‐HRP (HA1001; HuaBio Co. Ltd.) or goat polyclonal anti‐mouse IgG‐HRP (HA1006; HuaBio Co. Ltd.) were used as secondary antibodies. All chemiluminescent blots were imaged by an Image Quant LAS4000 mini (GE Healthcare, Chicago, IL, USA). Each experiment was repeated twice.

### 4.11 CUT&Tag Sequencing and Data Analysis

CUT&Tag libraries were constructed using the Hyperactive Universal CUT&Tag Assay Kit for Illumina (TD903, Vazyme, China). Briefly, the intact nuclei were isolated from 0.25 g of mycelia of each treatment and resuspended in 10 µL binding buffer with ConA beads. After incubating for 10 min at 25°C, the ConA bead–nucleus complex was pelleted by hopper magnet and resuspended in 50 µL of cold antibody buffer. Then, 2.5 µL of primary antibody of H3K9ac or H3K27ac (A7255, A7253, Abclonal) was added, and the reaction solution was incubated at 4°C overnight. The ConA bead–nucleus complex was pelleted again by hopper magnet and resuspended in 50 µL Dig‐wash buffer containing a secondary antibody (Goat Anti‐Rabbit IgG H&L (Abcam, #ab6702, 1:100 dilution). After incubation at 25°C for 60 min, the ConA bead–nucleus complex was pelleted and washed with 200 µL of Dig‐wash buffer three times. Subsequently, the complex was resuspended in 100 µL of Dig‐300 buffer containing 2 µL of pA/G‐Tn5 and kept for reaction at 25°C for 60 min. After that, the beads were pelleted and washed with 200 µL of Dig‐300 buffer three times. The beads were resuspended in 50 µL of Dig‐300 buffer containing 10 µL of 5× TTBL and incubated at 37°C for 60 min. Then, 5 µL of proteinase K, 100 µL of buffer L/B and 20 µL of DNA extract beads were added and incubated at 55°C for 10 min. The beads were pelleted and washed with 200 µL of WA buffer once and WB buffer twice. After the beads were air dried, the DNA fragments binding on beads were eluted with 22 µL of pure water, 20 µL of which was used for library preparation. The libraries were sequenced on an Illumina NovaSeq platform under the 2 × 150‐bp mode. Raw reads of CUT&Tag sequencing were processed to remove adapters using Cutadapt (v.4.1) [44]. The cleaned reads (≥20 bp) were aligned to the PH‐1 genome using Bowtie2 (v.2.4.5) [45]. Peaks were called using MACS2 (v2.2.7) [46] with ‘–keep‐dup all’ parameter for H3K9ac and H3K27ac. Coverage tracks with a bin size of 50 bp were calculated using the bamCoverage and RPKM normalized data. The generated datasets were visualized by pyGenomeTracks [47]. Each experiment was repeated twice.

### 4.12 Hi‐C Sequencing and Data Analysis

The mycelia of PH‐1 were fixed in a 1% (v/v) formaldehyde solution for 30 min at room temperature. The cross‐linking reaction was terminated by the addition of a glycine solution. The fixed mycelia were resuspended in 1 mL of lysis buffer and incubated on ice for 20 min. Nuclei were pelleted through centrifugation at 600 × g for 5 min at 4°C, followed by thorough washing with 1 mL of lysis buffer [48]. Once pelleted, the nuclei were resuspended in 400 µL of a restriction enzyme buffer and transferred to a safe lock tube. The chromatin was solubilized by the addition of dilute sodium dodecyl sulfate (SDS) and incubated at 65°C for 10 min. After quenching the SDS with Triton X‐100, overnight digestion was carried out using the restriction endonuclease MboI at 37°C on a rocking platform. The resulting cohesive ends were filled with a biotin marker to generate blunt ends, followed by ligation using T4 DNA ligase (New England Biolabs Inc.). DNA cross‐linking was then disrupted using proteinase K (Thermo Fisher, Waltham, MA), and DNA was purified through phenol‐chloroform extraction. Nonligated fragment ends carrying biotin labels were removed using T4 DNA polymerase. Subsequently, the DNA fragments were sheared by sonication to achieve a size range of 200–600 bp. The fragments were end‐repaired by a mixture of T4 DNA polymerase, T4 polynucleotide kinase, and Klenow DNA polymerase. Biotin‐labeled DNA fragments were purified through pulldown using streptavidin magnetic beads. The DNA fragment ends were then subjected to A‐tailing using Klenow DNA polymerase, followed by the addition of Illumina pairedend sequencing adapters using a ligation mix [49]. Finally, the Hi‐C libraries were amplified by PCR with 12–14 cycles and sequenced on an Illumina NovaSeq platform under the 2 × 150‐bp mode.

Raw Hi‐C reads were processed to remove adapters and low‐quality sequences. The cleaned Hi‐C reads were aligned to the PH‐1 genome using bwa (v.0.7.17) [50] with the parameters set to ‘‐A1 ‐B4 ‐E50 ‐L0’. The paired alignments were then processed using HiCExplorer (v.3.7.2) [51] to generate the contact matrices at the resolution of 1‐kb. Hi‐C matrices with different bin resolutions were generated using HicMergeMatrixBins. All matrices were normalized using HicNormalize, followed by Knight‐Ruiz matrix balancing using hicCorrectMatrix. Hi‐C matrices of different file formats were converted using hicConvertFormat. Observed/expected matrix was computed using hicTransform with ‘obs_exp_lieberman’ method. Jet‐like domains were called as previously described [18]. Hi‐C data from biological replicates were combined before data analysis.

## Author Contributions

Z.M. and Y.Z conceived and supervised the project. Y.Z., J.W., and Q.C. performed experiments. Y.Z., J.W., C.J., Y.C., Z.W., Z.M. analyzed the data. Y.Z., Z.W., and Z.M. wrote the manuscript.

## Conflicts of Interest

The authors declare no conflicts of interest.

## Supporting information




**Supporting File**: advs74762‐sup‐0001‐SuppMat.docx.

## Data Availability

ChIP‐seq data for Sge1 in PH‐1, RNA‐seq data for ΔFgSge1, Hi‐C data for ΔFgSge1, CUT&Tag data for H3K27ac and H3K9ac in ΔFgSge1 generated in this study have been deposited at the National Genomics Data Center BioProject database under accession number PRJCA041900. RNA‐seq and Hi‐C data for PH‐1 and ΔFgGcn5, and CUT&Tag data for H3K27ac and H3K9ac in PH‐1 and ΔFgGcn5 are publicly available at the National Center for Biotechnology Information BioProject database under accession number PRJNA995191 [18]. RNA‐seq data for ΔFgAda2 is publicly available at the National Genomics Data Center database under accession number CRA011377.

## References

[1] Y. Chen , J. Wang , N. Yang , et al., “Wheat Microbiome Bacteria Can Reduce Virulence of a Plant Pathogenic Fungus by Altering Histone Acetylation,” Nature Communications 9, no. 1 (2018): 3429, 10.1038/s41467-018-05683-7. PMC6109063 30143616

[2] K. Y. Seong , M. Pasquali , X. Zhou , et al., “Global Gene Regulation by Fusarium Transcription Factors Tri6 and Tri10 Reveals Adaptations for Toxin Biosynthesis,” Molecular Microbiology 72, no. 2 (2009): 354–367, 10.1111/j.1365-2958.2009.06649.x. 19320833

[3] T. Ma , L. Zhang , M. Wang , et al., “Plant Defense Compound Triggers Mycotoxin Synthesis by Regulating H2B ub1 and H3K4 me2/3 Deposition,” New Phytologist 232, no. 5 (2021): 2106–2123, 10.1111/nph.17718. 34480757 PMC9293436

[4] Y. Wang , T. Cao , D. Liu , et al., “The pH Signaling Pathway Pal/PacC Regulates Fungal Growth, Stress Responses, and Mycotoxin Biosynthesis in Fusarium Graminearum,” Crop Health 3, no. 1 (2025): 17, 10.1007/s44297-025-00054-3. 41649649 PMC12825994

[5] C. Xu , J. Wang , Y. Zhang , et al., “The Transcription Factor FgStuA Regulates Virulence and Mycotoxin Biosynthesis via Recruiting the SAGA Complex in Fusarium Graminearum,” New Phytologist 240, no. 6 (2023): 2455–2467, 10.1111/nph.19297. 37799006

[6] W. Jonkers , Y. Dong , K. Broz , and H. C. Kistler , “The Wor1‐Like Protein Fgp1 Regulates Pathogenicity, Toxin Synthesis and Reproduction in the Phytopathogenic Fungus Fusarium Graminearum,” PLoS Pathogens 8, no. 5 (2012): 1002724, 10.1371/journal.ppat.1002724. PMC3364952 22693448

[7] G. Huang , H. Wang , S. Chou , X. Nie , J. Chen , and H. Liu , “Bistable Expression of WOR1 , a Master Regulator of White–opaque Switching in Candida albicans,” Proceedings of the National Academy of Sciences 103, no. 34 (2006): 12813–12818, 10.1073/pnas.0605270103. PMC1540355 16905649

[8] F. W. Yu , X. P. Zhang , M. H. Yu , Y. N. Yin , and Z. H. Ma , “The Potential Protein Kinase A (Pka) Phosphorylation Site Is Required for the Function of FgSge1 in Fusarium Graminearum,” World Journal of Microbiology and Biotechnology 31, no. 9 (2015): 1419–1430, 10.1007/s11274-015-1894-2. 26130440

[9] Z. Han , R. Yu , D. Xiong , and C. Tian , “A Sge1 Homolog in Cytospora Chrysosperma Governs Conidiation, Virulence and the Expression of Putative Effectors,” Gene 778 (2021): 145474, 10.1016/j.gene.2021.145474. 33549711

[10] C. B. Michielse , R. van Wijk , L. Reijnen , et al., “The Nuclear Protein Sge1 of Fusarium Oxysporum Is Required for Parasitic Growth,” PLoS Pathogens 5, no. 10 (2009): 1000637, 10.1371/journal.ppat.1000637. PMC2762075 19851506

[11] J. Jiang , J. Lu , D. Lu , et al., “Investigation of the Acetylation Mechanism by GCN5 Histone Acetyltransferase,” PLoS ONE 7, no. 5 (2012): 36660, 10.1371/journal.pone.0036660. PMC3344931 22574209

[12] A. Hawar , W. Chen , T. Zhu , et al., “The Histone Acetyltransferase GCN5 Regulates Floral Meristem Activity and Flower Development in Arabidopsis,” The Plant Cell 37, no. 6 (2025): koaf135, 10.1093/plcell/koaf135. 40413778

[13] C. Servet , N. Conde e Silva , and D. X. Zhou , “Histone Acetyltransferase AtGCN5/HAG1 Is a Versatile Regulator of Developmental and Inducible Gene Expression in Arabidopsis,” Molecular Plant 3, no. 4 (2010): 670–677, 10.1093/mp/ssq018. 20457643

[14] C. Servet , M. Benhamed , D. Latrasse , W. Kim , M. Delarue , and D. X. Zhou , “Characterization of a Phosphatase 2C Protein as an Interacting Partner of the Histone Acetyltransferase GCN5 in Arabidopsis,” Biochimica et Biophysica Acta (BBA)—Gene Regulatory Mechanisms 1779, no. 6‐7 (2008): 376–382, 10.1016/j.bbagrm.2008.04.007. 18498779

[15] C. Weiste and W. Dröge‐Laser , “The Arabidopsis Transcription Factor bZIP11 Activates Auxin‐Mediated Transcription by Recruiting the Histone Acetylation Machinery,” Nature Communications 5 (2014): 3883, 10.1038/ncomms4883. 24861440

[16] H. C. Paes , L. D. S. Derengowski , L. D. F. Peconick , et al., “A Wor1‐Like Transcription Factor Is Essential for Virulence of Cryptococcus Neoformans,” Frontiers in Cellular and Infection Microbiology 8 (2018): 369, 10.3389/fcimb.2018.00369. 30483479 PMC6243373

[17] S. Zhou and C. Wu , “Comparative Acetylome Analysis Reveals the Potential Roles of Lysine Acetylation for DON Biosynthesis in Fusarium Graminearum,” BMC Genomics 20, no. 1 (2019): 841, 10.1186/s12864-019-6227-7. 31718553 PMC6852988

[18] W. Shao , J. Wang , Y. Zhang , et al., “The Jet‐Like Chromatin Structure Defines Active Secondary Metabolism in Fungi,” Nucleic Acids Research 52, no. 9 (2024): 4906–4921, 10.1093/nar/gkae131. 38407438 PMC11109943

[19] L. Howe , C. E. Brown , T. Lechner , and J. L. Workman , “Histone Acetyltransferase Complexes and Their Link to Transcription,” Critical Reviews in Eukaryotic Gene Expression 9, no. 3‐4 (1999): 231–243, 10.1615/critreveukargeneexpr.v9.i3-4.80. 10651240

[20] Q. Gu , Y. Wang , X. Zhao , et al., “Inhibition of Histone Acetyltransferase GCN5 by a Transcription Factor FgPacC Controls Fungal Adaption to Host‐Derived Iron Stress,” Nucleic Acids Research 50, no. 11 (2022): 6190–6210, 10.1093/nar/gkac498. 35687128 PMC9226496

[21] D. W. Brown , M. Busman , and R. H. Proctor , “Fusarium Verticillioides SGE1 Is Required for Full Virulence and Regulates Expression of Protein Effector and Secondary Metabolite Biosynthetic Genes,” Molecular Plant‐Microbe Interactions 27, no. 8 (2014): 809–823, 10.1094/mpmi-09-13-0281-r. 24742071

[22] P. Santhanam and B. P. Thomma , “Verticillium Dahliae Sge1 Differentially Regulates Expression of Candidate Effector Genes,” Molecular Plant‐Microbe Interactions 26, no. 2 (2013): 249–256, 10.1094/mpmi-08-12-0198-r. 22970788

[23] T. Caspari , “Onset of Gluconate‐H+ Symport in Schizosaccharomyces Pombe Is Regulated by the Kinases Wis1 and Pka1, and Requires the gti1+ Gene Product,” Journal of Cell Science 110, no. Pt 20 (1997): 2599–2608, 10.1242/jcs.110.20.2599. 9372449

[24] M. Wang , L. Wu , Y. Mei , et al., “Host‐Induced Gene Silencing of Multiple Genes of Fusarium Graminearum Enhances Resistance to Fusarium Head Blight in Wheat,” Plant Biotechnology Journal 18, no. 12 (2020): 2373–2375, 10.1111/pbi.13401. 32436275 PMC7680546

[25] R. Zhang , K. An , Y. Gao , et al., “The Transcription Factor CAMTA2 Interacts with the Histone Acetyltransferase GCN5 and Regulates Grain Weight in Wheat,” The Plant Cell 36, no. 12 (2024): 4895–4913, 10.1093/plcell/koae261. 39321218 PMC11638106

[26] J. R. Dixon , S. Selvaraj , F. Yue , et al., “Topological domains in mammalian genomes identified by analysis of chromatin interactions,” Nature 485, no. 7398 (2012): 376–380, 10.1038/nature11082. 22495300 PMC3356448

[27] I. Jerkovic , G. Cavalli , et al., “Understanding 3D genome organization by multidisciplinary methods,” Nature Reviews Molecular Cell Biology 22, no. 8 (2021): 511–528, 10.1038/s41580-021-00362-w. 33953379

[28] A. R. Barutcu , P. G. Maass , J. P. Lewandowski , et al., “A TAD boundary is preserved upon deletion of the CTCF‐rich Firre locus,” Nature Communications 9, no. 1 (2018): 1444, 10.1038/s41467-018-03614-0. PMC5899154 29654311

[29] G. Olley , M. Ansari , H. Bengani , et al., “BRD4 interacts with NIPBL and BRD4 is mutated in a Cornelia de Lange‐like syndrome,” Nature Genetics 50, no. 3 (2018): 329–332, 10.1038/s41588-018-0042-y. 29379197 PMC6469577

[30] J. Huang , W. Dai , D. Xiao , et al., “Acetylation‐Dependent SAGA Complex Dimerization Promotes Nucleosome Acetylation and Gene Transcription,” Nature Structural & Molecular Biology 29, no. 3 (2022): 261–273, 10.1038/s41594-022-00736-4. 35301489

[31] Y. Yu , F. Zhao , Y. Yue , Y. Zhao , and D. X. Zhou , “Lysine Acetylation of Histone Acetyltransferase Adaptor Protein ADA2 Is a Mechanism of Metabolic Control of Chromatin Modification in Plants,” Nature Plants 10, no. 3 (2024): 439–452, 10.1038/s41477-024-01623-0. 38326652

[32] J. Wang , C. Xu , Q. Sun , et al., “Post‐Translational Regulation of Autophagy Is Involved in Intra‐Microbiome Suppression of Fungal Pathogens,” Microbiome 9, no. 1 (2021): 131, 10.1186/s40168-021-01077-y. 34092253 PMC8182927

[33] Y. Yun , Z. Liu , J. Zhang , W. B. Shim , Y. Chen , and Z. Ma , “The MAPKK FgMkk1 of Fusarium Graminearum Regulates Vegetative Differentiation, Multiple Stress Response, and Virulence via the Cell Wall Integrity and High‐Osmolarity Glycerol Signaling Pathways,” Environmental Microbiology 16, no. 7 (2014): 2023–2037, 10.1111/1462-2920.12334. 24237706

[34] Y. Yun , Z. Liu , Y. Yin , et al., “Functional Analysis of the Fusarium Graminearum Phosphatome,” New Phytologist 207, no. 1 (2015): 119–134, 10.1111/nph.13374. 25758923

[35] H. Wang , Y. Chen , T. Hou , Y. Jian , and Z. Ma , “The Very Long‐Chain Fatty Acid Elongase FgElo2 Governs Tebuconazole Sensitivity and Virulence in Fusarium Graminearum,” Environmental Microbiology 24, no. 11 (2022): 5362–5377, 10.1111/1462-2920.16212. 36111363

[36] F. Yu , Q. Gu , Y. Yun , et al., “The TOR Signaling Pathway Regulates Vegetative Development and Virulence in Fusarium Graminearum,” New Phytologist 203, no. 1 (2014): 219–232, 10.1111/nph.12776. 24684168

[37] K. Kaufmann , J. M. Muiño , M. Østerås , L. Farinelli , P. Krajewski , and G. C. Angenent , “Chromatin Immunoprecipitation (ChIP) of Plant Transcription Factors Followed by Sequencing (ChIP‐SEQ) or Hybridization to Whole Genome Arrays (ChIP‐CHIP),” Nature Protocols 5, no. 3 (2010): 457–472, 10.1038/nprot.2009.244. 20203663

[38] S. Chen , “Ultrafast One‐Pass FASTQ Data Preprocessing, Quality Control, and Deduplication Using fastp,” iMeta 2, no. 2 (2023): 107, 10.1002/imt2.107. PMC10989850 38868435

[39] T. L. Bailey , “STREME: Accurate and Versatile Sequence Motif Discovery,” Bioinformatics 37, no. 18 (2021): 2834–2840, 10.1093/bioinformatics/btab203. 33760053 PMC8479671

[40] D. Kim , J. M. Paggi , C. Park , C. Bennett , and S. L. Salzberg , “Graph‐Based Genome Alignment and Genotyping with HISAT2 and HISAT‐Genotype,” Nature Biotechnology 37, no. 8 (2019): 907–915, 10.1038/s41587-019-0201-4. PMC7605509 31375807

[41] S. Anders , P. T. Pyl , and W. Huber , “HTSeq—A Python Framework to Work with High‐Throughput Sequencing Data,” Bioinformatics 31, no. 2 (2015): 166–169, 10.1093/bioinformatics/btu638. 25260700 PMC4287950

[42] M. I. Love , W. Huber , and S. Anders , “Moderated Estimation of Fold Change and Dispersion for RNA‐seq Data with DESeq2,” Genome Biology 15, no. 12 (2014): 550, 10.1186/s13059-014-0550-8. 25516281 PMC4302049

[43] F. Ramírez , F. Dündar , S. Diehl , B. A. Grüning , and T. Manke , “deepTools: a Flexible Platform for Exploring Deep‐Sequencing Data,” Nucleic Acids Research 42, no. Web Server issue (2014): W187–W191, 10.1093/nar/gku365. 24799436 PMC4086134

[44] A. Kechin , U. Boyarskikh , A. Kel , and M. Filipenko , “cutPrimers: a New Tool for Accurate Cutting of Primers from Reads of Targeted Next Generation Sequencing,” Journal of Computational Biology 24, no. 11 (2017): 1138–1143, 10.1089/cmb.2017.0096. 28715235

[45] B. Langmead and S. L. Salzberg , “Fast Gapped‐Read Alignment with Bowtie 2,” Nature Methods 9, no. 4 (2012): 357–359, 10.1038/nmeth.1923. 22388286 PMC3322381

[46] Y. Zhang , T. Liu , C. A. Meyer , et al., “Model‐Based Analysis of ChIP‐Seq (MACS),” Genome Biology 9, no. 9 (2008): R137, 10.1186/gb-2008-9-9-r137. 18798982 PMC2592715

[47] L. Lopez‐Delisle , L. Rabbani , J. Wolff , et al., “pyGenomeTracks: Reproducible Plots for Multivariate Genomic Datasets,” Bioinformatics 37, no. 3 (2021): 422–423, 10.1093/bioinformatics/btaa692. 32745185 PMC8058774

[48] S. Zhao , Y. S. Yan , Q. P. He , et al., “Comparative Genomic, Transcriptomic and Secretomic Profiling of Penicillium Oxalicum HP7‐1 and Its Cellulase and Xylanase Hyper‐Producing Mutant EU2106, and Identification of Two Novel Regulatory Genes of Cellulase and Xylanase Gene Expression,” Biotechnology for Biofuels 9 (2016): 203, 10.1186/s13068-016-0616-9. 27688806 PMC5035457

[49] C. X. Li , L. Liu , T. Zhang , X. M. Luo , J. X. Feng , and S. Zhao , “Three‐Dimensional Genome Map of the Filamentous Fungus Penicillium Oxalicum,” Microbiology Spectrum 10, no. 3 (2022): 0212121, 10.1128/spectrum.02121-21. PMC9241887 35499317

[50] H. Li and R. Durbin , “Fast and Accurate Short Read Alignment with Burrows–Wheeler Transform,” Bioinformatics 25, no. 14 (2009): 1754–1760, 10.1093/bioinformatics/btp324. 19451168 PMC2705234

[51] J. Wolff , L. Rabbani , R. Gilsbach , et al., “Galaxy HiCExplorer 3: A Web Server for Reproducible Hi‐C, Capture Hi‐C and Single‐Cell Hi‐C Data Analysis, Quality Control and Visualization,” Nucleic Acids Research 48, no. W1 (2020): W177–W184, 10.1093/nar/gkaa220. 32301980 PMC7319437

