# Supplementary material for: The Transcription Factor FgSge1 Harnesses the SAGA Complex to Activate Mycotoxin Biosynthesis and Fungal Virulence
Source: Adv Sci (Weinh). 2026 Mar 12;13(29):e18558. doi: 10.1002/advs.202518558 (PMC13205826; doi:10.1002/advs.202518558)
Supplement: Supplementary file 1 — Supporting File: advs74762‐sup‐0001‐SuppMat.docx. [file ADVS-13-e18558-s001.docx]

**Figure S1.** Phylogenetic distribution, sequence identity, and protein length of FgSge1 homologs across diverse taxa. The maximum-likelihood phylogenetic tree (left) shows the evolutionary relationships of FgSge1-related proteins across archaea, bacteria, protists, oomycetes, plants, metazoa, and fungi, with branch lengths scaled by MYA (million years ago). The heatmap (middle) displays sequence identity (0-100%) relative to FgSge1, and the bar chart (right) shows the length distribution of these proteins. For fungal taxa, lifestyle categories (biotroph, hemibiotroph, necrotroph, and non-pathogens) are color-coded to highlight functional context.





**Figure S2.** FgSge1 regulates DON toxin biosynthesis and virulence. (A) Colony morphology of PH-1, the mutant and complementation strain of FgSge1 on CM, MM, PDA. (B) DON production in PH-1, mutant and complementation strain. Each strain was determined for DON production after growth in TBI for seven days. Data presented are the mean ± standard errors from three repeated experiments (n = 3). Different letters represent statistically significant differences according to the one-way ANOVA test (*P* < 0.05) followed by Fisher’s least significant difference (LSD) test. (C) Virulence of the PH-1, mutant and complementation strain on wheat head. Infected wheat heads were examined after point inoculated with fresh mycelia of each strain for 15 d. The inoculated site on each wheat head was labeled with a black dot. The experiment was repeated three times independently with similar results. (D) Virulence of the PH-1, mutant and complementation strain on corn silk. Corn silks were examined after 4 d of inoculation with mycelial plugs of each tested strain. The experiment was repeated three times independently with similar results. (E) Virulence of the PH-1, mutant and complementation strain on wheat coleoptiles. Representative images were taken 3 d after inoculation. The experiment was repeated three times independently with similar results. (F) FgSge1 mutant showed the same sensitive to SDS and CR compared with the wild type. Colony morphology was examined after growth on CM medium with or without 0.02% SDS or 0.02% CR for 2 days. (G) Strains penetrating cellophane membrane experiment. Fresh mycelia agar plates of PH-1, ΔFgSge1, and the complemented strain were placed on the surface of cellophane membranes overlaid on CM medium and incubated at 25°C for 2 days (Before). After removing the cellophane membranes with attached agar plates, incubation continued for an additional 2 days. Observation of hyphal growth on the surface of the petri dishes determined whether the cellophane membrane had been penetrated (After). (H) Comparison of transcription levels of genes associated with penetration between wild-type PH-1 and ΔFgSge1 in RNA-seq analysis. Functional annotation of penetrating genes: galactosidase synthesis genes (*FGRAMPH1_01G12939* and *FGRAMPH1_01G12467*), glucosidase synthesis genes (FGRAMPH1_01G06317, *FGRAMPH1_01G21153* and *FGRAMPH1_01G27623*), glycoside hydrolase genes (*FGRAMPH1_01G00503* and *FGRAMPH1_01G25059*), lipase releasing free fatty acid gene *FGRAMPH1_01G19051*, xyloside xylohydrolase gene *FGRAMPH1_01G10847*, and glucosidase gene *FGRAMPH1_01G19195*.


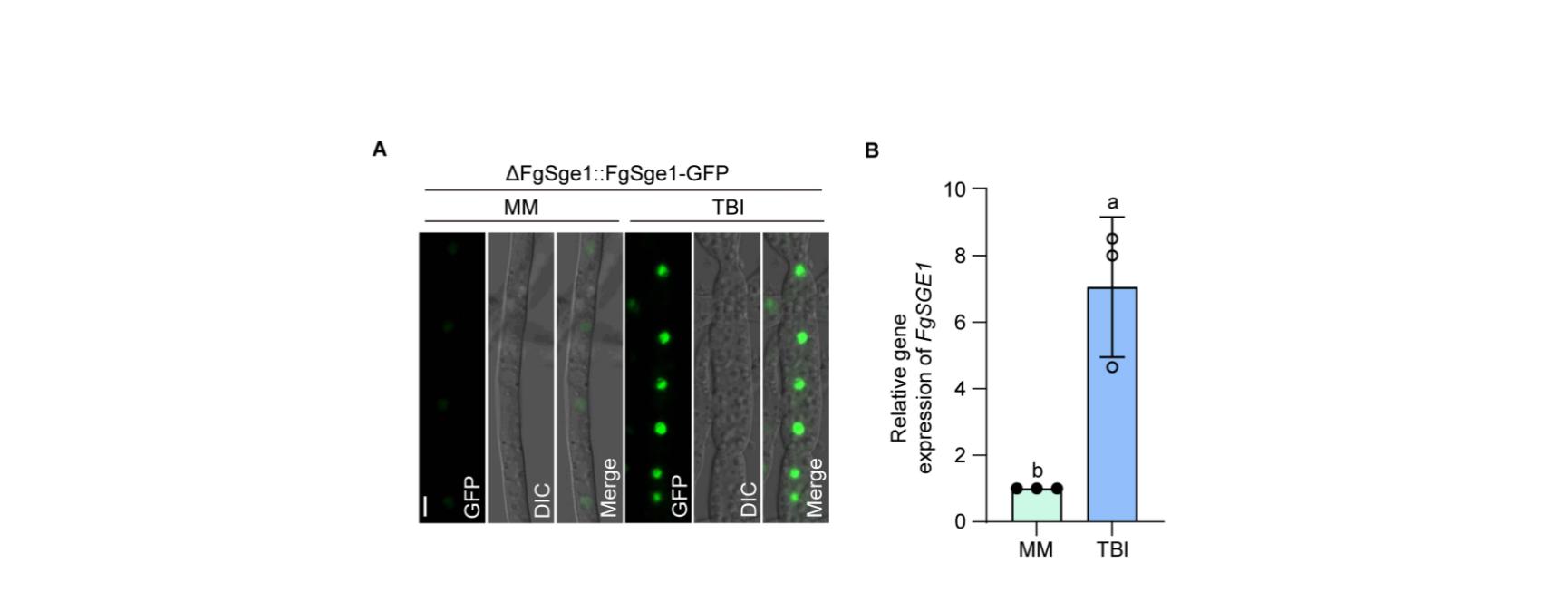


**Figure S3.** *FgSGE1* transcription is induced in TBI medium. (A) Confocal microscopy observation on FgSge1-GFP localization in mycelia of ΔFgSge1:: FgSge1-GFP in the liquid MM and TBI medium. Bar = 10 μm. (B) The relative transcription level of *FgSGE1* in the liquid MM and TBI. Mean and standard deviation were estimated with data from three biological replicates (marked with dark and white dots, n = 3). Different letters represent statistically significant differences according to the one-way ANOVA test (*P* < 0.05) followed by Fisher’s least significant difference (LSD) test.





**Figure S4.** The N- and C-terminal truncation mutants of FgSge1 interacted with FgAda2 in yeast two-hybrid (Y2H) assay. Yeast cells grew on minimal synthetic defined (SD) medium lacking leucine (L), tryptophan(T) (SD-L/T) and medium lacking leucine (L), tryptophan(T), histidine (H) and adenine (A) (SD-L-T-H-A).





**Figure S5.** FgSge1 regulates 3D chromatin conformation in *F. graminearum*. (A) The heatmap of chromatin interactions at the chromosome level. Log_2_ ratio plot between 10 kb Hi-C matrices of ΔFgSge1 and WT. Red and blue indicate contacts are higher in ΔFgSge1 and WT, respectively. (B) Average intra-chromosomal contact frequency as a function of genomic distance for PH-1 and ΔFgSge1. (C) Modelled 3D structure of *F. graminearum* genome. The contact matrix was computed with a resolution of 20 kb.

**Table S1. PCR primers used in this study.**

| Primer | Primer Sequence (5’-3’) | Relevant characteristics |
| --- | --- | --- |
| Sge1-KO-UP-F | CATACATGCCCCCTTATCGAG | PCR primers for amplification of *FgSGE1* upstream fragment to construct FgSge1 deletion mutants |
| Sge1-KO-UP-R | CAAAATAGGCATTGATGTGTTGACCTCCGTGAGTGTTGTGTGAGTGTTG |  |
| Sge1-KO-DOWN-F | CTCGTCCGAGGGCAAAGGAATAGAGTAGGGGGAAATTCTTTTTAATTAG | PCR primers for amplification of *FgSGE1* downstream fragment to construct FgSge1 deletion mutants |
| Sge1-KO-DOWN-R | GCAATATACATAGTAACAGC |  |
| Sge1-C-UP-F | CATTGCAGAGTACAGATGCAT | PCR primers for amplifciation *FgSGE1* upstream fragment for construct FgSge1 complement strain |
| Sge1-C-UP-R | CAGCTCCTCGCCCTTGCTCACTTGTAATTGGGCGTGATACC |  |
| GFP-F | GTGAGCAAGGGCGAGGAGCTG | PCR primers for amplification of *GFP* fragment |
| GFP-R | TTACTTGTACAGCTCGTCCATG |  |
| G418-F | CATGGACGAGCTGTACAAGTAAGGAGGTCAACACATCAATGC | PCR primers for amplification of *G418* resistance gene |
| G418-R | TCAGAAGAACTCGTCAAGAAG |  |
| Sge1-C-DOWN-F | CTTCTTGACGAGTTCTTCTGAGCTAGGGTGAGGGGAAATTCTT | PCR primers for amplifciation *FgSGE1* downstream fragment for construct FgSge1 complement strain |
| Sge1-C-DOWN-R | GCAATATACATAGTAACAGC |  |
| Sge1-GST-F | TCCCCGAATTCCCGGGTCGACATGGCAAATTCAGTGCTCACAG | PCR primers for amplification of *FgSGE1* CDS fragment for the construction of FgSge1-GST vector |
| Sge1-GST-R | GATGCGGCCGCTCGAGTCGACTTGTAATTGGGCGTGATAC |  |
| Motif-bio-F | TAAAGTTTTAAAGTTTTAAAGTTT | PCR primers for amplification of the cis-element for EMSA assays |
| Motif-bio-R | AAACTTTTAAACTTTTAAACTTTA |  |
| Motif-MUT-bio-F | TAAAGCCTTAAAGCCTTAAAGCCT | PCR primers for amplificatipmn of the mutant cis-element for EMSA assays |
| Motif-MUT-bio-R | AGGCTTTTAAGGCTTTTAAGGCTTA |  |
| SproGFP-upF | CATACATGCCCCCTTATCGAG | PCR primers for amplification of *FgSGE1* upstream fragment for the construction of sge1 native promoter tagged GFP |
| SproGFP-upR | CAGCTCCTCGCCCTTGCTCACCATGTGAGTGTTGTGTGAGTGTTG |  |
| SproGFP-DF | CTTCTTGACGAGTTCTTCTGAGGGGAAATTCTTTTTAATTAG | PCR primers for amplification of *FgSGE1* downstream fragment for the construction of sge1 native promoter tagged GFP |
| SproGFP-DR | GCAATATACATAGTAACAGC |  |
| Sge1-BD -F | TCAGAGGAGGACCTGCATATGATGGCAAATTCAGTGCTCACAG | PCR primers for amplification of *FgSGE1* CDS fragment for the construction of pGBKT7-FgSge1 vector |
| Sge1-BD-R | TTCGGCCTCCATGGCCATATGTCATTGTAATTGGGCGTGATAC |  |
| Sge1-N-BD-F | TCAGAGGAGGACCTGCATATGATGTATGAGGGTTATATCAGG | PCR primers for amplification of N- terminal of *FgSGE1* CDS fragment for the construction of pGBKT7-N-FgSge1 vector |
| Sge1-N-BD-R | TCGACGGATCCCCGGGAATTCTTAATAGTAGCTCACGAGGTGG |  |
| Sge1-C-BD-F | TCAGAGGAGGACCTGCATATGATGACTGTCGATGACGTGAGG | PCR primers for amplification of C- terminal of *FgSGE1* CDS fragment for the construction of pGBKT7-N-FgSge1 vector |
| Sge1-C-BD-R | TCGACGGATCCCCGGGAATTCTCATTGTAATTGGGCGTGATAC |  |
| Gcn5-AD-F | GTACCAGATTACGCTCATATGATGTCAGACGAAAACGGC | PCR primers for amplification of *FgGCN5* CDS fragment for the construction of pGADT7-FgGcn5 vector |
| Gcn5-AD-R | ATGCCCACCCGGGTGGAATTCTTTCTCAGGCTCCAGGTGAG |  |
| Ada2-AD-F | GTACCAGATTACGCTCATATGATGGGTGTTATTAGAAAG | PCR primers for amplification of *FgADA2* CDS fragment for the construction of pGADT7-FgAda2 vector |
| Ada2-AD-R | ATGCCCACCCGGGTGGAATTCTCATTGGCATGGGGTGCAC |  |
| Gcn5-RFP-F | ACTCACTATAGGGCGAATTGGGTACTCAAATTGGTTATGTCAGACGAAAGTAAGTCC | PCR primers for amplification of *FgGCN5* ORF fragment for the construction of FgGcn5-RFP strain |
| Gcn5-RFP-R | CATGAACTCCTTGATGACGTCCTCGGAGGAGGCCATTTTCTCAGGCTCCAGGTGAG |  |
| Ada2-RFP-F | ACTCACTATAGGGCGAATTGGGTACTCAAATTGGTTGTCATATTCGGGGAGTTC | PCR primers for amplification of *FgADA*2 ORF fragment for the construction of FgAda2-RFP strain |
| Ada2-RFP-R | CATGAACTCCTTGATGACGTCCTCGGAGGAGGCCATTTGGCATGGGGTGCACGCCTG |  |
| Flag-Ada2-UP-F | GTGATATCATGAGCGTACTTTG | PCR primers for amplification *FgAda2* upstream fragment for the construction of Flag-FgAda2 strain |
| Flag-Ada2-UP-R | AATAGGCATTGATGTGTTGACCTCCACTGTCGGTTGCGATGTCGG |  |
| Flag-Ada2-DOWN-F | GATATCGATTACAAGGATGACGATGACAAGATGGGTGTTATTAGAAAGAAAAC | PCR primers for amplification *FgADA2* downstream fragment for the construction of Flag-FgAda2 strain |
| Flag-Ada2-DOWN-R | CGAATGGCTTGGCCTTTTGC |  |
| Flag-Gcn5-UP-F | AAAACGAATACCTTCCTCTG | PCR primers for amplification *FgGCN5* upstream fragment for the construction of Flag-FgGcn5 strain |
| Flag-Gcn5-UP-R | AATAGGCATTGATGTGTTGACCTCCGATTGGTGCGGGCTCAACCC |  |
| Flag-Gcn5-DOWN-F | GATATCGATTACAAGGATGACGATGACAAGATGTCAGACGAAAGTAAGTC | PCR primers for amplification *FgGCN5* downstream fragment for the construction of Flag-FgGcn5 strain |
| Flag-Gcn5-DOWN-R | CCTCAGGCGTAAGGTACTGG |  |
| Sge1-KO-UP-F | CTGGGCTTGGAGGAGAAGAGA | PCR primers for amplification of *FgADA2* upstream fragment to construct FgAda2 deletion mutants |
| Sge1-KO-UP-R | GCTCCTTCAATATCACTAGTACTGTCGGTTGCGATGTCGGGC |  |
| Sge1-KO-DOWN-F | CTAAACCAAAGCATCAGGCCTCCAAGATGAAATTGAACTG | PCR primers for amplification of *FgADA2* downstream fragment to construct FgAda2 deletion mutants |
| Sge1-KO-DOWN-R | GGGTTAATTTGTCTTTCTTGC |  |
| Nat-F | ACTAGTGATATTGAAGGAGC | PCR primers for amplification of nourseothricin resistance gene |
| Nat-R | AGGCCTGATGCTTTGGTTTAG |  |
| Hph-F | GGAGGTCAACACATCAATGCCTATT | PCR primers for amplification of hygromycin resistance gene |
| Hph-R | CTATTCCTTTGCCCTCGGACG |  |
| GPDA-F | CGTCCGAGGGCAAAGGAATAGTGCGGAGAGACGGACGGACG | PCR primers for amplification of GPDA promoter and Flag fragment |
| GPDA-Flag-R | CTTGTCATCGTCATCCTTGTAATCGATATCATGATCTTTATAATCACCGTCATGGTCTTTGTAGTCCATGGTGATGTCTGCTCAAGCGG |  |
| Tri4-RT-F | CATGCAGAGTGGTACGACTG | PCR primers for the expression levels analysis of *TRI4* gene |
| Tri4-RT-R | CATACTTTTGACCATGCATG |  |
| Tri5-RT-F | TAGTCTAACTAACACACAGG | PCR primers for the expression levels analysis of *TRI5* gene |
| Tri5-RT-R | AAACTGCCGGCCATCACAT |  |
| Tri6-RT-F | CATGCAGAGTGGTACGACTG | PCR primers for the expression levels analysis of *TRI6* gene |
| Tri6-RT-R | TGACCATGCATGGGTCTCGG |  |
| Tri10-RT-F | CGTCCACTGAACGAGGCGTACAG | PCR primers for the expression levels analysis of *TRI10* gene |
| Tri10-RT-R | AAAGACGGGAAGGAATGGGC |  |
| Tri14-RT-F | CAGCCCAAGGCCTACTGTTG | PCR primers for the expression levels analysis of *TRI14* gene |
| Tri14-RT-R | GACTGATCCTCGGTATTGTG |  |
| But1-RT-F | TGCACTGCACCGCTTGTAAC | PCR primers for the expression levels analysis of *BUT1* gene |
| But1-RT-R | TCCTCAGTGGACACAACTG |  |
| But2-RT-F | CTTGTAACCAACATGCAAC | PCR primers for the expression levels analysis of *BUT2* gene |
| But2-RT-R | TCCTCAGTGGACACAACTG |  |
| GFP-RT-F | TGCAGCTCGCCGACCACTAC | PCR primers for the expression levels analysis of *GFP* gene |
| GFP-RT-R | GCTCGTCCATGCCGAGAGTG |  |
| act-RT-F | ATCCACGTCACCACTTTCAA | PCR primers for the expression levels analysis of *ACTIN* gene |
| act-RT-R | TGCTTGGAGATCCACATTTG |  |
